# Listerin-Dependent Nascent Protein Ubiquitination Relies on Ribosome Subunit Dissociation

**DOI:** 10.1016/j.molcel.2013.04.015

**Published:** 2013-06-06

**Authors:** Sichen Shao, Karina von der Malsburg, Ramanujan S. Hegde

**Affiliations:** 1 MRC Laboratory of Molecular Biology, Francis Crick Avenue, Cambridge CB2 0QH, UK

## Abstract

Quality control of defective mRNAs relies on their translation to detect the lesion. Aberrant proteins are therefore an obligate byproduct of mRNA surveillance and must be degraded to avoid disrupting protein homeostasis. These defective translation products are thought to be ubiquitinated at the ribosome, but the mechanism of ubiquitin ligase selectivity for these ribosomes is not clear. Here, we in vitro reconstitute ubiquitination of nascent proteins produced from aberrant mRNAs. Stalled 80S ribosome-nascent chain complexes are dissociated by the ribosome recycling factors Hbs1/Pelota/ABCE1 to a unique 60S-nascent chain-tRNA complex. The ubiquitin ligase Listerin preferentially recognizes 60S-nascent chains and triggers efficient nascent chain ubiquitination. Interfering with Hbs1 function stabilizes 80S complexes, precludes efficient Listerin recruitment, and reduces nascent chain ubiquitination. Thus, ribosome recycling factors control Listerin localization, explaining how translation products of mRNA surveillance are efficiently ubiquitinated while sparing translating ribosomes.

## Highlights

• Nascent protein ubiquitination on stalled ribosomes has been reconstituted in vitro
• Ubiquitination on separated 60S subunit is strongly preferred over intact 80S ribosome
• Nascent chain inside a 60S subunit cues stable binding of ubiquitin ligase Listerin
• Subunit dissociation by ribosome recycling factors enables ligase recruitment

## Introduction

Cells have various surveillance mechanisms to detect and degrade defective mRNAs (Doma and Parker, 2007). These defects can include inappropriate internal polyadenylation, absence of an in-frame stop codon, internal damage or break, extensive secondary structure, and premature stop codons. Detection of these lesions typically relies on unsuccessful translation of the mRNA, leading to a stalled ribosome (Maquat et al., 2010; Shoemaker and Green, 2012). Stalled translation complexes are therefore diagnostic of mRNA lesions and can trigger degradation of the mRNA to avoid its repeated use. Hence, continued production of potentially defective proteins is avoided.

However, the requirement for at least one round of translation during mRNA surveillance means defective protein production cannot be entirely avoided. Heavy traffic through mRNA surveillance in organisms with complex transcriptomes and extensive regulation can generate a substantial burden of incomplete or otherwise defective protein byproducts (Drummond and Wilke, 2009; Ingolia et al., 2011; Isken and Maquat, 2008). Efficient degradation of these products is important for maintaining protein homeostasis and avoiding disease (Balch et al., 2008; Drummond and Wilke, 2009). Thus, recent work has investigated the pathway for degrading nascent protein products of stalled ribosomes.

Studies by Ito-Harashima et al. (2007) first demonstrated that “nonstop” mRNAs lacking an in-frame stop codon produce proteins that are efficiently degraded by the proteasome. Translation of the poly(A) tail (which encodes polylysine) was postulated to trigger protein destabilization. Indeed, a 12 residue polybasic coding segment was sufficient to induce ribosome stalling and efficient proteasome-mediated degradation of the partially synthesized product (Ito-Harashima et al., 2007). Although the ubiquitin ligase Not4 was initially implicated in this pathway (Dimitrova et al., 2009), work by Bengtson and Joazeiro (2010) found an essential role for the ubiquitin ligase Ltn1 in degrading nonstop translation products and fragments resulting from internal polybasic stalls. The observation that Ltn1 is ribosome associated led to a model in which polybasic sequences trigger Ltn1-mediated ubiquitination of the nascent chain for downstream degradation.

Recent work from Brandman et al. (2012) and Defenouillère et al. (2013) showed that Ltn1 is part of a ribosome quality control complex (RQC) containing Tae2, Rqc1, and the Cdc48 complex. Each of these factors is required for degradation of polybasic-mediated stalled proteins (Bengtson and Joazeiro, 2010; Brandman et al., 2012; Verma et al., 2013; Defenouillère et al., 2013). The RQC copurified with 60S ribosomal subunits that contained ubiquitinated proteins in a Ltn1-dependent manner (Brandman et al., 2012; Defenouillère et al., 2013). Cdc48 deficiency caused nondegraded nascent chains to accumulate as ubiquitinated peptidyl-tRNAs on ribosomes (Verma et al., 2013; Defenouillère et al., 2013). The level of ubiquitinated products that accumulate in Cdc48 mutant cells depended partially on Ltn1 (Verma et al., 2013). These findings suggest that translational stalls lead to Ltn1-mediated nascent chain ubiquitination, dissociation of the ribosome, and Cdc48-dependent extraction and degradation of the nascent chain.

Stalled ribosome dissociation involves three factors: Hbs1, Dom34 (Pelota in mammals), and Rli1 (ABCE1 in mammals). The GTPase Hbs1 forms a complex with Dom34 and interacts with the A site of stalled or vacant ribosomes in a GTP-dependent manner (Becker et al., 2011; Pisareva et al., 2011; Shoemaker et al., 2010). GTP hydrolysis by Hbs1 leads to its dissociation concomitant with a conformational change in Dom34. This permits recruitment of Rli1, which uses its ATPase activity to drive subunit dissociation and recycling of the ribosomal subunits (Becker et al., 2012; Pisareva et al., 2011; Shoemaker and Green, 2011). In addition, ribosome recycling facilitates mRNA degradation in two ways. First, the Hbs1:Dom34 complex may stimulate endonucleolytic cleavage of the mRNA to initiate its degradation (Doma and Parker, 2006; Lee et al., 2007; Passos et al., 2009). Second, vacating the mRNA of ribosomes may permit its access to the exosome (Tsuboi et al., 2012; van Hoof et al., 2002). Thus, ribosome recycling is increasingly appreciated to play a key role in mRNA surveillance pathways (Tsuboi et al., 2012).

The observation that RQC pull-downs copurify the 60S subunit without an associated 40S (Brandman et al., 2012) suggests some relationship between ribosome dissociation and Ltn1 recruitment. Indeed, deletion of Dom34 leads to at least partial stabilization of stalled translation products (Tsuboi et al., 2012; Verma et al., 2013), although the interpretation is complicated by simultaneous effects on mRNA stability. Furthermore, the order in which these events occur and the basis of Ltn1 specificity for stalled ribosomes remain obscure. Puzzlingly, Ltn1 is found coassociated with 60S subunits (Bengtson and Joazeiro, 2010; Brandman et al., 2012), while undegraded nascent chains that accumulate in strains deficient for RQC function are primarily free or in 80S complexes (Bengtson and Joazeiro, 2010; Verma et al., 2013; Defenouillère et al., 2013). Thus, it is unclear where Ltn1 initially meets its nascent chain clients to mediate their ubiquitination.

To begin addressing these mechanistic issues, we have developed an in vitro system that recapitulates ubiquitination of stalled nascent chains in a reaction dependent on Listerin (the mammalian ortholog of yeast Ltn1). The manipulability of the in vitro system allowed us to order the events leading to Listerin recruitment and ubiquitination, establish the role played by ribosome recycling factors, and identify the basis of Listerin specificity. These results provide a framework for ribosome-associated quality control and an experimental system for mechanistic dissection of this process.

## Results

### Reconstitution of Ribosome-Associated Nascent Chain Ubiquitination In Vitro

In vitro translation of an mRNA truncated in the coding region generates a ribosome-nascent chain complex (RNC) stalled at the 3′ end of the message. Such RNCs are often employed as putatively stable intermediates to study protein translation, targeting, and translocation (Perara et al., 1986). As part of our earlier studies of protein translocation and cytosolic quality control (Hessa et al., 2011), we made RNCs of prion protein in reticulocyte lysate. As expected, this nascent chain was ubiquitinated in the cytosol when released from the ribosome with puromycin (see Figure S1A online). The same polypeptide also became ubiquitinated as a RNC, although the pattern was noticeably different (Figure S1A). Depletion of hydrophobic-binding factors from the cytosol to remove key components of cytosolic quality control (Hessa et al., 2011) prevented ubiquitination of the released nascent chain (Figure S1B). However, RNC ubiquitination was unchanged in the depleted extract (Figure S1B).

Sucrose gradient analysis of in vitro-produced RNCs showed a proportion of ubiquitinated nascent chains sedimenting with the ribosomal fractions (Figure S1C). An unrelated nascent chain (GFP) that is typically not subjected to cytosolic quality control (Dimitrova et al., 2009; Hessa et al., 2011) was also ubiquitinated as an RNC and cosedimented with ribosomes (Figure S1D). These initial observations suggested that nascent chains might be ubiquitinated on the ribosome in vitro by a pathway distinct from cytosolic quality control pathways for released nascent chains. Because truncated mRNAs in vivo are subject to translation-dependent mRNA and protein degradation, we realized our in vitro observations may provide a route to dissect the mechanism of nascent chain ubiquitination during mRNA surveillance.

**Figure 1:**
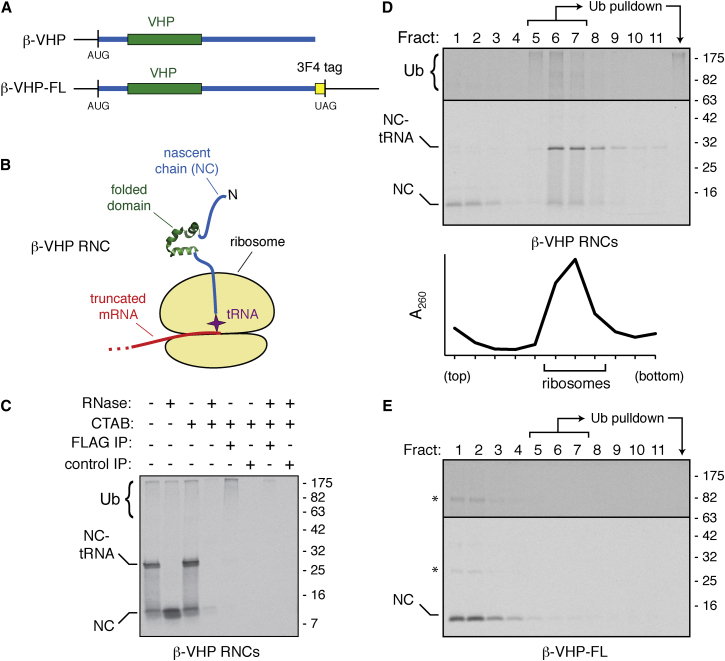
Nascent Chain-tRNAs on Stalled Ribosomes Are Ubiquitinated In Vitro (A) Line diagram of truncated and full-length (FL) β-VHP transcripts. (B) Schematic diagram of a stalled β-VHP ribosome-nascent chain complex (RNC). (C) In vitro translation reactions of β-VHP RNCs containing FLAG-tagged ubiquitin were sequentially treated with RNase, precipitated with CTAB, and/or immunoprecipitated with anti-FLAG or control antibodies as indicated. The positions of the nascent chain (NC) containing or lacking a tRNA are indicated, along with ubiquitinated products (Ub). (D and E) Translation reactions of β-VHP or β-VHP-FL containing His-tagged ubiquitin were separated on 10%–50% sucrose gradients. Each fraction was immunoprecipitated with an antibody against the substrate and visualized by autoradiography. The upper portion of the gel was exposed 4-fold longer than the lower portion to visualize ubiquitinated products. An A_260_ trace is shown to indicate ribosomal fractions. An aliquot of pooled fractions 5–7 was also subject to immobilized Co^2+^ pull-downs to recover His-tagged ubiquitinated products (last lane). See also Figure S1.

To investigate this process in detail, we designed a simplified RNC substrate containing a small tightly folded domain outside the ribosomal exit tunnel tethered to the ribosomal P site tRNA by a 54 residue unstructured linker (Figures 1A and 1B). The unstructured region was taken from the cytosolic part of Sec61β, while the 35 residue autonomously folding villin head piece (VHP) served as the folded domain (McKnight et al., 1996). This truncated RNC, termed β-VHP, ends with valyl-tRNA, whose ester bond is exceptionally stable to spontaneous hydrolysis (Hentzen et al., 1972). This facilitates maintenance of the tRNA during sample processing and SDS-PAGE.

Translation of the truncated β-VHP mRNA in reticulocyte lysate resulted in a ^35^S-labeled primary translation product plus a high molecular weight smear (Figure 1C). The primary translation product represents β-VHP covalently attached to the peptidyl-tRNA, as evidenced by a decrease in molecular weight upon treatment with RNase (Figure 1C) or puromycin (Figure S2A) and selective precipitation by the nucleic acid precipitant cetyl trimethylammonium bromide (CTAB) (Figure 1C). This product quantitatively migrated with ribosomes through a sucrose gradient (Figure 1D), and immunoprecipitation of an HA-tagged version coprecipitated the ribosome (Figure S2B).

The high-molecular-weight smear represents ubiquitinated β-VHP-tRNA on the basis of its CTAB precipitation (Figure 1C), sedimentation with the ribosomal fractions through a sucrose gradient (Figure 1D), and immunoprecipitation with antibodies against tagged-ubiquitin (Figures 1C and 1D) and substrate (Figure 1D, Figure S2C). Analysis with mutant ubiquitin suggested that K48 chains were being added (Figure S2D), consistent with eventual targeting to the proteasome (Chau et al., 1989). The ubiquitination of β-VHP-tRNA saturated when only ∼1%–2% of ribosomes are programmed (Figure S2E), suggesting that this pathway is relatively low abundance. Importantly, full-length β-VHP transcript containing a termination codon (β-VHP-FL) produced a nonubiquitinated primary translation product that did not cosediment with ribosomes (Figure 1E).

**Figure 2:**
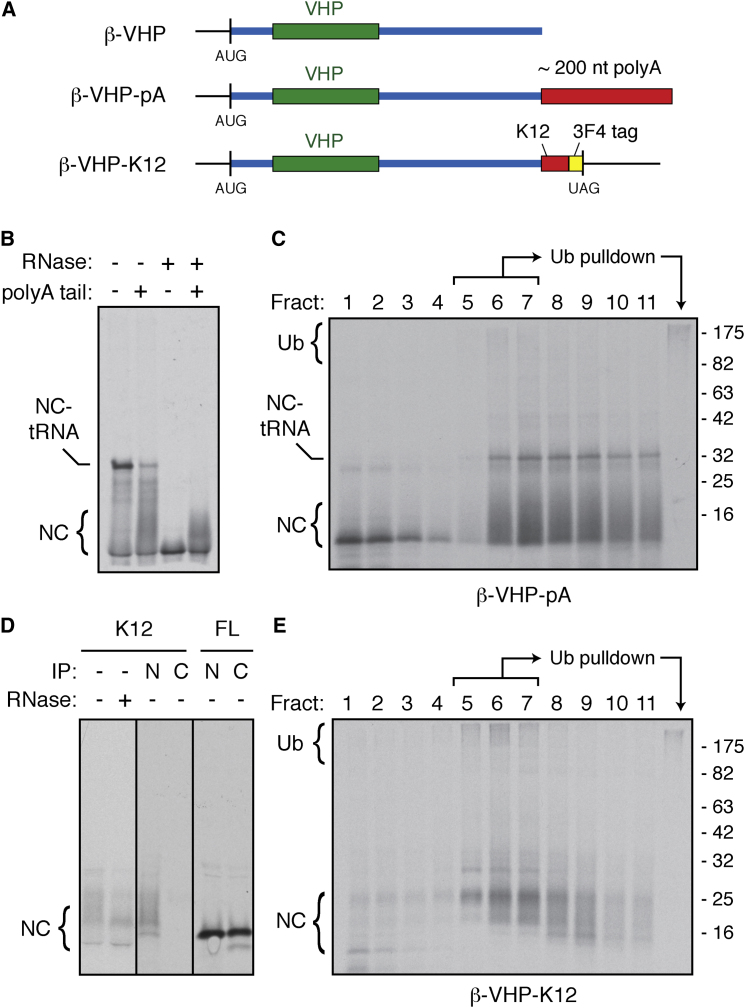
Internally Stalled Ribosome-Nascent Chains Are Ubiquitinated (A) Line diagrams of transcripts coding for truncated β-VHP, truncated β-VHP with a poly(A) tail (β-VHP-pA), and full-length β-VHP with a polybasic tract (β-VHP-K12). (B) Truncated β-VHP with or without a poly(A) tail was translated and subject to RNase treatment. The migration of nascent chain-tRNA (NC-tRNA) and nascent chain (NC) is indicated. (C) Analysis of β-VHP-pA as in Figure 1C. (D) β-VHP-K12 was translated and subject to RNase treatment or immunoprecipitations with antibodies against either the N or C terminus of the protein. Immunoprecipitations of β-VHP-FL translations are shown for comparison. (E) Analysis of β-VHP-K12 as in Figure 1C. See also Figure S2.

Translational stalls induced by three other methods all led to varying degrees of ribosome-tethered ubiquitinated nascent chains (Figure 2). In the first, we added an ∼200 nt poly(A) tail to truncated β-VHP to produce a nonstop mRNA (β-VHP-pA) whose translation should read into the poly(A) tail (Figure 2A). Translation of β-VHP-pA produced a heterogeneous set of products, some of which contained a tethered tRNA, as evidenced by their sensitivity to RNase digestion (Figure 2B). Note that the lysyl-tRNA at the end of these truncated products is particularly labile to hydrolysis (Hentzen et al., 1972) and is not preserved efficiently during electrophoresis. The polypeptides from β-VHP-pA were larger than the β-VHP product but too small to have translated to the end of an ∼200 nucleotide poly(A) tail. This suggested stalling of ribosomes after partial translation into the poly(A) tail. Indeed, a substantial proportion of the translation products cosedimented with ribosomes on a sucrose gradient (Figure 2C), as did a high-molecular-weight smear. Ubiquitin pull-downs of the ribosomal fractions showed that this high-molecular-weight smear represents ubiquitinated nascent chains.

Similar results were obtained with stalling mediated by an internal polylysine domain (β-VHP-K12) (Figure 2A). As with β-VHP-pA, β-VHP-K12 produced a heterogeneous set of translation products, some of which were sensitive to RNase digestion. Importantly, the translation products lacked an epitope tag C-terminal to the polylysine stretch (Figure 2D), confirming that translation stalled prior to reaching the termination codon. The stalled β-VHP-K12 products were primarily observed in the ribosomal fractions. Ubiquitin pull-downs from these fractions verified that the high-molecular-weight smear represented polyubiquitinated nascent chains (Figure 2E).

Finally, a stem loop placed in the β-VHP transcript also led to partial stalling, cosedimentation with the ribosomes, and ubiquitinated products in the ribosomal fractions (Figure S2F). In this case, stalling was incomplete compared to expectations from yeast studies with this stem loop (Becker et al., 2011; Doma and Parker, 2006), perhaps because mammalian translation complexes have higher helicase activity. Nevertheless, the stalled product, identified by the attached tRNA and cosedimentation with ribosomes, was polyubiquitinated, as evidenced by recovery of high-molecular-weight nascent chains in ubiquitin pull-downs. Thus, nascent chain-tRNAs stalled at the 3′ end of a message, in the poly(A) tail of a nonstop mRNA, internally at a polybasic stretch, or physically by a stem loop can all be ubiquitinated at the ribosome in vitro.

### Nascent Chain Ubiquitination via Listerin Recruitment to Stalled RNCs

**Figure 3:**
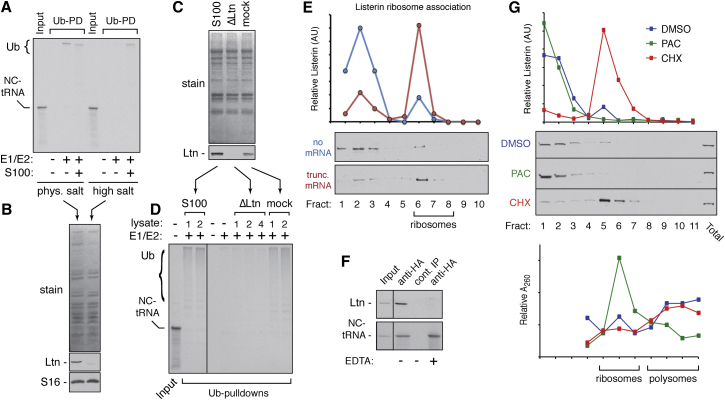
Listerin Is Recruited to Stalled RNCs to Mediate Ubiquitination (A) β-VHP RNCs purified under physiological or high salt conditions (input) were incubated with the indicated components plus His-tagged ubiquitin and ATP, followed by pull-down of the ubiquitinated products via the His-tag (Ub-PD). (B) RNCs from (A) were stained for total protein or immunoblotted for Listerin (Ltn) and ribosomal protein S16. (C) S100 cytosol was immunodepleted with anti-Listerin (ΔLtn) or nonimmune (mock) antibody and stained for total proteins and immunoblotted for Listerin. (D) High salt-washed RNCs (input) were subjected to ubiquitination assays (as in A) with the indicated components and different relative amounts of the lysates from (C). (E) Translation reactions containing no mRNA or a truncated mRNA (β-VHP) were separated on 10%–50% sucrose gradients and individual fractions analyzed by immunoblotting for Listerin. The Listerin blot and its quantification are shown. (F) In vitro translation reactions of HA-tagged β-VHP RNCs (input) were immunoprecipitated with control or anti-HA antibodies in the presence of either 2 mM MgCl_2_ or 10 mM EDTA and analyzed by immunoblotting to detect Listerin (top) and the translation product (bottom). (G) HEK293T cells pretreated for 1 hr with 200 nM pactamycin, 50 μg/mL cycloheximide, or nothing were harvested and lysates fractionated by 10%–50% sucrose gradients. Fractions were analyzed for Listerin (quantified below the blots) and A_260_ absorbance. An aliquot of the total lysate was also included in the blots (last lane). See also Figure S3.

Since ubiquitination is delayed by 12–15 min after synthesis of the nascent chain (Figure S3A), we could isolate the RNCs at an early time point and analyze ubiquitination in a subsequent reaction. “Posttranslational” ubiquitination on RNCs was observed upon incubation with cytosol and energy (Figure S3B). The cytosol requirement for ubiquitination could be entirely replaced with purified E1 and E2 enzymes (Figure 3A). An E2 enzyme screen showed that Ubc4/5 homologs (UbcH5 and UbcH6) were preferred (Figure S3C), consistent with their role in degradation of short-lived proteins (Seufert and Jentsch, 1990). The requirement for only E1 and E2 enzymes suggested that the E3 ubiquitin ligase responsible for nascent chain ubiquitination copurifies with the RNC.

Given the proposed role for the yeast E3 ligase Ltn1 in ubiquitination of nascent chains on the ribosome (Bengtson and Joazeiro, 2010; Brandman et al., 2012), we investigated the involvement of its mammalian ortholog Listerin (Chu et al., 2009). RNCs isolated through a high salt gradient did not contain Listerin (Figure 3B) and lost ubiquitination activity (Figure 3A). A ribosome-free cytosolic fraction (S-100) fully restored ubiquitination to salt-washed RNCs (Figure 3A). S-100 derived from an RNC translation reaction was partially depleted of both Listerin and RNC ubiquitination activity (Figures S3D and S3E). Passing S-100 over resin containing affinity-purified anti-Listerin antibody also depleted Listerin (Figure 3C) and RNC ubiquitination activity (Figure 3D). Thus, Listerin appears to be recruited to RNCs from a soluble pool and mediates nascent chain ubiquitination.

Sucrose gradient analysis showed that Listerin shifted from being primarily soluble to primarily ribosome-associated after translation of a truncated mRNA (Figure 3E). Affinity purification of RNCs via the nascent chain copurified Listerin in an EDTA-sensitive manner (Figure 3F). EDTA disrupts ribosome structure by Mg^2+^chelation (Klein et al., 2004), suggesting an indirect nascent chain-Listerin interaction via the ribosome. Because translation of a truncated mRNA was required for effective Listerin recruitment to the ribosomal fraction, the far more abundant endogenous ribosomes and polysomes are apparently not efficient targets for Listerin.

Analysis of cultured cell lysates by sucrose gradients (Figure 3G) also showed a portion of Listerin sedimenting in the ribosomal fractions. This population was not observed if cells were pretreated for 1 hr with the translation initiation inhibitor pactamycin. By contrast, nearly all of the cell’s Listerin was ribosome associated when translational stalling was enforced with cycloheximide. These data suggest that in vivo, as seen in vitro, Listerin is recruited to ribosomes in a translation-dependent manner, and this recruitment is enhanced by ribosome stalling.

### 60S-Nascent Chain Complexes Are Preferential Targets for Ubiquitination

**Figure 4:**
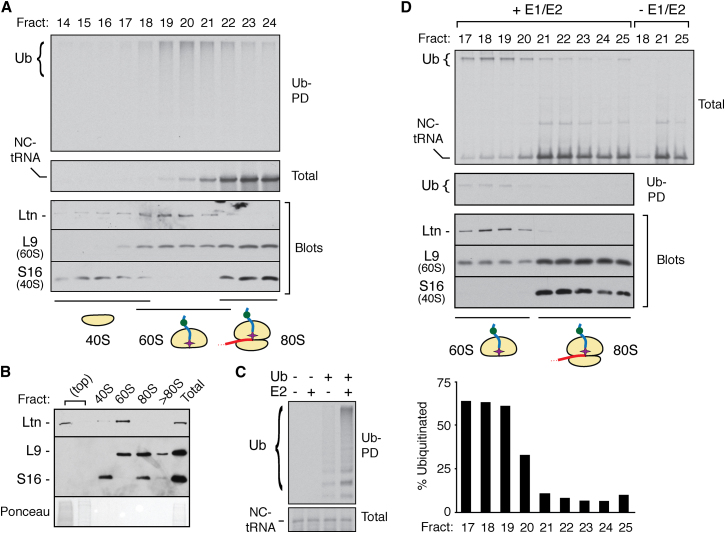
60S-Nascent Chain-tRNA Complex Is the Selective Target for Ubiquitination (A) β-VHP RNCs were produced in the presence of His-tagged ubiquitin and separated on a 10%–30% sucrose gradient. Individual fractions were analyzed for ubiquitinated products via His pull-down (Ub-PD), the nascent chain (total), Listerin, and ribosomal proteins of the 60S (L9) and 40S (S16) subunits. Fractions with 40S, 60S, and 80S complexes are shown. (B) HEK293T cells pretreated for 1 hr with 50 μg/mL cycloheximide were harvested and fractionated on a 10%–30% sucrose gradient and analyzed by immunoblotting for Listerin and ribosomal subunits. Peak fractions of key complexes are displayed. (C) β-VHP was translated in a ubiquitin- and E2-deficient fractionated translation extract (Fr-RRL) replenished with His-tagged ubiquitin (Ub) and 250 nM UbcH5a (E2) as indicated. The samples were analyzed directly (lower panel) or subject to His pull-downs of ubiquitinated products (top). (D) β-VHP RNCs produced in Fr-RRL were separated on a 10%–30% sucrose gradient and individual fractions incubated with or without E1 and E2 enzymes plus His-Ubiquitin and ATP. The samples were analyzed directly (total) and after ubiquitin pull-down (Ub-PD). The proportion of ubiquitinated substrate in each fraction was quantified (bottom). Blots of the fractions show the positions of Listerin and the ribosomal proteins. See also Figure S4.

What is the basis of Listerin specificity for stalled but not elongating nascent chains? One clue came from the observation that both Listerin and polyubiquitinated nascent chains consistently migrate close to the leading edge of ribosomal fractions through sucrose gradients (Figure 1D). Furthermore, yeast studies had found Ltn1 cosedimenting with 60S subunits (Bengtson and Joazeiro, 2010), and pull-downs of RQC copurified only 60S ribosomal proteins (Brandman et al., 2012; Defenouillère et al., 2013). Analysis of our ubiquitination reactions on higher-resolution gradients revealed clear cosedimentation of 60S ribosomal subunits, Listerin, and the polyubiquitinated nascent chain-tRNA (Figure 4A). In these fractions, a high proportion (50% or more) of the nascent chain-tRNA was ubiquitinated. By contrast, few if any polyubiquitinated products were seen in the 80S fractions beyond that expected from the tail of the 60S peak. Similar results were seen for poly(A), K12, and stem-loop-mediated stalled RNCs (Figures S4A–S4C). Furthermore, cultured cells containing cycloheximide-induced translational stalls showed nearly all Listerin cofractionating with 60S subunits (Figure 4B). Thus, Listerin is found with ubiquitinated nascent chain-tRNAs on 60S subunits, not 80S ribosomes.

This could be explained if stalled 80S RNCs are split by ribosome recycling factors (Becker et al., 2012; Pisareva et al., 2011; Shoemaker and Green, 2011) to generate 60S-nascent chain complexes that are the target for ubiquitination. Hence, ubiquitinated nascent chains would preferentially be observed on 60S subunits because ubiquitination competence is only acquired after splitting. To test this idea, we produced RNCs in a ubiquitination-deficient lysate (Hessa et al., 2011) depleted of the requisite E2s and ubiquitin (Figure 4C). The products were separated on a high-resolution sucrose gradient, and each fraction was incubated with E1, E2, ubiquitin, and ATP to identify ubiquitination-competent complexes. 60S-nascent chain complexes contained Listerin and were ∼10-fold better substrates for ubiquitination than 80S RNCs (Figure 4D). Identical results were observed when translation time was used to uncouple nascent chain synthesis from ubiquitination (Figure S4D). Thus, stalled 80S RNCs seem to bind Listerin poorly and do not support efficient nascent chain ubiquitination. Conversion of 80S RNCs to 60S-nascent chain complexes favors stable Listerin binding, resulting in efficient ubiquitination.

### Bipartite Recognition of 60S-Nascent Chains by Listerin

Because 60S-nascent chain complexes are a unique molecular species never observed during the normal translation cycle, their selective targeting by Listerin would explain how a ribosome-associating E3 ligase could avoid promiscuously ubiquitinating translating nascent chains. Yet specificity cannot reside in either the nascent chain, which is identical in the 80S RNC, or the recycled 60S subunit, which is produced as part of normal translation termination. This suggests that their combination provides the specificity for Listerin recruitment. To test this, we compared Listerin recruitment to 60S-nascent chain complexes, 60S subunits lacking nascent chains, and free nascent chain-tRNA.

**Figure 5:**
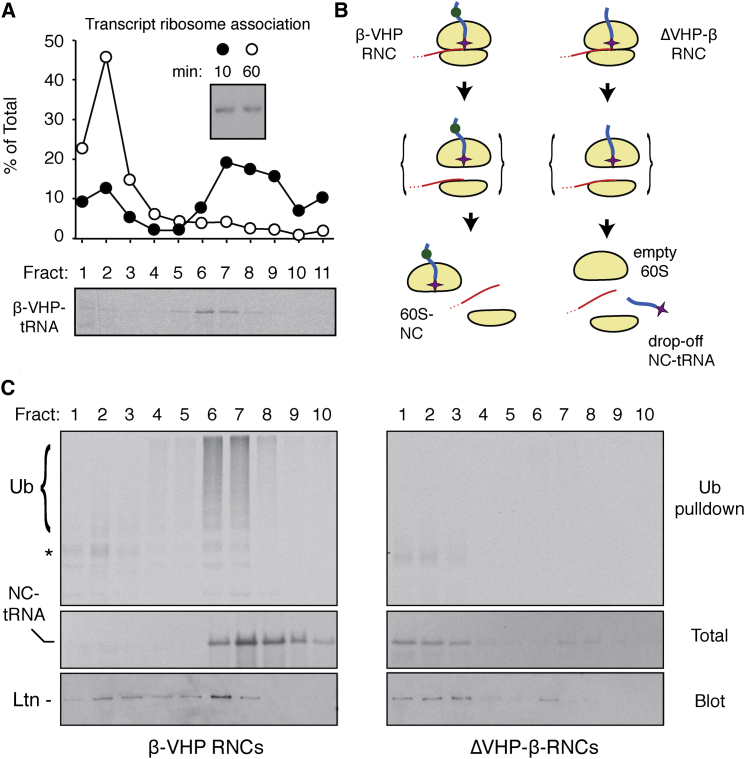
Bipartite Recognition of 60S-Nascent Chain Substrates by Listerin (A) ^33^P-labeled β-VHP transcript was used to program a 60 min translation reaction, with 100 μM aurin tricarboxylic acid added at 10 min to inhibit initiation. Samples taken at 10 and 60 min were analyzed by gel electrophoresis and autoradiography (inset) or separated on a 10%–50% sucrose gradient and quantified by scintillation counting (graph). Ribosomes migrate in fractions 6–9. A matched reaction containing unlabeled transcript and ^35^S-methionine was analyzed in parallel for migration of the protein product (lower panel). (B) Scheme to generate 60S subunits containing or lacking a nascent chain after ribosome splitting. (C) Translation reactions of β-VHP (left) or ΔVHP-β (right) containing His-tagged ubiquitin were separated on a 10%–50% sucrose gradient. Individual fractions were analyzed by autoradiography (total) or immunoblotting for Listerin, or subjected to pull-downs for ubiquitinated products. See also Figure S5.

We sought to generate each of these products in our translation reactions to analyze their respective interactions. We first confirmed efficient ribosome splitting in our system by illustrating that radiolabeled truncated β-VHP transcript is almost completely released to the soluble fraction at the end of the translation reaction (60 min; Figure 5A). An aliquot of the reaction taken at 10 min showed that the transcript was primarily ribosome associated at the time further initiation was inhibited with aurin tricarboxylic acid (Figure 5A). Gel analysis of the transcript before and after the reaction verified that it remained intact throughout (Figure 5A, inset).

Importantly, β-VHP-tRNA in this reaction remained in the ribosomal fraction, consistent with it being trapped in the 60S subunit by the folded VHP domain and attached tRNA (Figures 5A–5C). A matched construct lacking the folded VHP domain (ΔVHP−β) was not ribosome associated by the end of the translation (Figures 5B and 5C). The presence of an intact tRNA on ΔVHP−β (verified by RNase digestion; data not shown) confirmed that it had dropped off the ribosome upon splitting. This is consistent with earlier work on ribosome recycling showing that a short nascent chain-tRNA drops off the ribosome upon subunit splitting (Pisareva et al., 2011; Shoemaker et al., 2010). Thus, ribosome splitting of β-VHP RNCs generates 60S-nascent chain complexes, while splitting of ΔVHP−β RNCs produces an equivalent number of matched vacant 60S subunits (see diagram, Figure 5B).

Immunoblotting for Listerin across the sucrose gradients of β-VHP and ΔVHP−β reactions showed reduced ribosomal recruitment in the latter (Figure 5C). Pull-down of ubiquitinated products from these gradient samples showed that ribosome-associated β-VHP-tRNA was polyubiquitinated while free β-tRNA was not (Figure 5C). Thus, upon ribosome splitting, a vacant 60S subunit does not recruit Listerin as effectively as a 60S-nascent chain complex.

Because Listerin comigrates with the dropped-off ΔVHP−β-tRNA (Figure 5C), we asked whether they were coassociated. For this, we generated drop-off products of a 3X-FLAG-tagged ΔVHP−β and analyzed their interactions by affinity purification and mass spectrometry. The tRNA-dependent interacting partners of affinity-captured ΔVHP−β-tRNA were selectively eluted with RNase, after which the remaining products were eluted with FLAG peptide (Figure S5A). All products were analyzed by mass spectrometry. Listerin was not among the identified proteins and could not be detected by blotting (Figure S5B). Instead, the major associating proteins were the eEF1 complex and valyl-tRNA synthetase, both of which can bind the valyl-tRNA at the end of the nascent chain. No specific interacting proteins were recovered if the samples were pretreated with RNase (Figure S5A). Listerin was also not detectable by crosslinking analysis of radiolabeled ΔVHP−β-tRNA drop-off products (Figure S5C).

These results suggest that Listerin does not efficiently bind to either a naked nascent chain-tRNA or vacant 60S subunits; only when the nascent chain-tRNA is within the 60S subunit is Listerin recruited to polyubiquitinate the nascent chain. This “bipartite” dependence for Listerin interaction explains why it would not interfere with 60S biogenesis or ribosome joining.

### Ribosome Splitting Is a Prerequisite for Listerin-Mediated Ubiquitination

**Figure 6:**
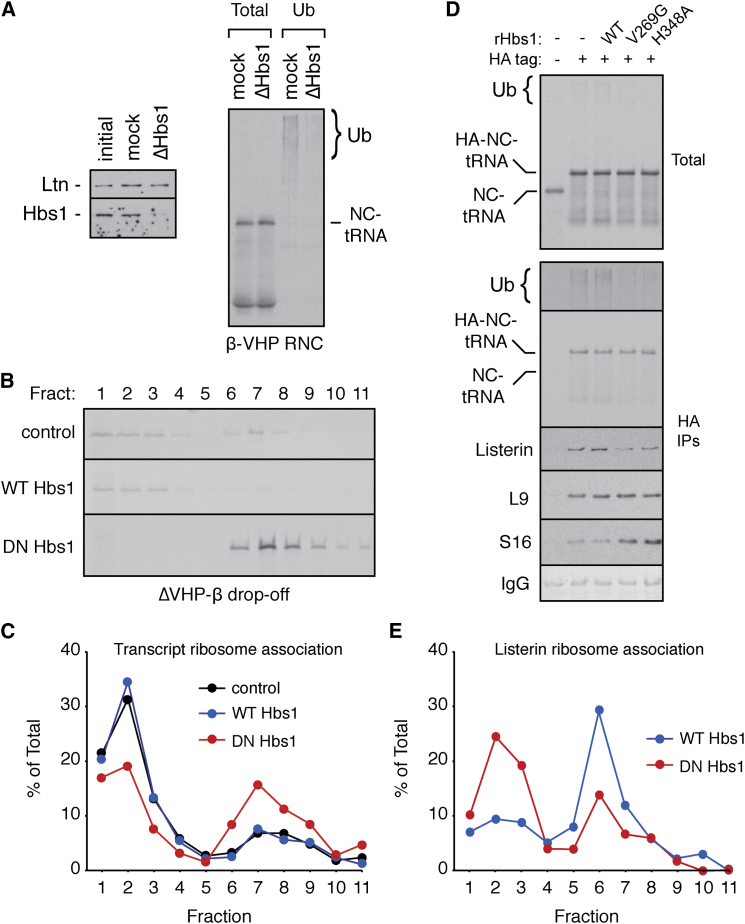
Ribosome Subunit Dissociation Precedes Listerin-Mediated Ubiquitination (A) Translation extracts were passed over a control (mock) or Pelota-conjugated resin (ΔHbs1) and blotted for Listerin and Hbs1 (left panel). β-VHP was translated in mock or ΔHbs1 lysates and analyzed directly (total) or subjected to ubiquitin pull-downs (Ub) before analysis. (B) ΔVHP-β was translated in the absence (control) or presence of 5-fold excess wild-type (WT) or dominant-negative (DN) H348A Hbs1 and separated on a 10%–50% gradient, and each fraction was analyzed by autoradiography. (C) Translation reactions of ^33^P-labeled β-VHP transcript in the absence (control) or presence of excess WT or DN Hbs1 were separated on a 10%–50% gradient, and each fraction was quantified by scintillation counting. (D) β-VHP transcripts lacking or containing an N-terminal 3× HA-tag were translated in the absence or presence of WT or two different dominant-negative Hbs1 mutants. The samples were analyzed directly (total) or after affinity purification via the HA tag (HA IPs). Translation products were analyzed by autoradiography, with a long exposure of the upper part of the gel to detect ubiquitinated species. Immunoblotting was used to detect copurifying Listerin and ribosomal proteins L9 and S16. (E) β-VHP was translated with an excess of WT or DN Hbs1, separated on a 10%–50% gradient, and the relative amount of Listerin in each fraction was quantified by blotting. See also Figure S6.

The preferential binding of Listerin to 60S-nascent chain complexes suggests that splitting of stalled 80S RNCs is a prerequisite for Listerin recruitment and hence ubiquitination. To test this idea, we depleted Hbs1 (by ∼80%–90%) from translation extracts using a column immobilized with its binding partner Pelota (Figure 6A) and tested the effect on stalled RNC ubiquitination. Stalled nascent chains produced from truncated β-VHP were markedly reduced in their ubiquitination (Figure 6A). Similar results were seen for stalling mediated by β-VHP-pA and β-VHP-K12 (Figure S6A). Recombinant Hbs1 purified from mammalian cells (Figure S6B) partially restored ubiquitination to the depleted extracts, while a GTPase-deficient mutant of Hbs1 did not (Figure S6C). Thus, Hbs1 activity appears to stimulate efficient nascent chain ubiquitination.

This conclusion was validated by illustrating that GTPase-deficient mutants of Hbs1 act as dominant negatives that trap the stalled 80S complex, preclude efficient Listerin recruitment, and inhibit nascent chain ubiquitination. Inhibition of ribosome splitting by the GTPase mutant H348A (Hbs1-DN) was evidenced by the failure of ΔVHP-β nascent peptides to drop off the ribosome (Figure 6B). Furthermore, release of radiolabeled β-VHP transcript from 80S RNCs that normally accompanies ribosome recycling (Figure 5A) was reduced by Hbs1-DN, but not wild-type Hbs1 (Figure 6C). Analysis of β-VHP ubiquitination showed ∼90% inhibition of ubiquitination by Hbs1-DN, but not wild-type Hbs1 (Figure 6D). A different GTPase mutant (V269G) showed ∼60% inhibition of ubiquitination. Thus, preventing ribosome splitting via a dominant-negative strategy inhibited nascent chain ubiquitination.

Pull-downs via stalled HA-tagged β-VHP showed that RNCs prepared with Hbs1-DN contained ∼3-fold less Listerin and more 40S subunit than parallel reactions containing wild-type Hbs1 or lacking any recombinant protein (Figure 6D). Analysis of the Hbs1-DN reactions on a sucrose gradient showed that when 80S RNCs are trapped at the presplitting stage, Listerin is not recruited to them as efficiently as reactions where splitting is allowed to occur (Figure 6E). The residual Listerin observed in the ribosomal fractions of the Hbs1-DN samples comigrated with 60S subunits on separate high-resolution gradients (Figure S6D). Thus, stable recruitment of Listerin to the nascent chain cannot occur until ribosome splitting by the Hbs1:Pelota system. In the absence of Listerin recruitment, nascent chains are not ubiquitinated efficiently.

## Discussion

**Figure 7:**
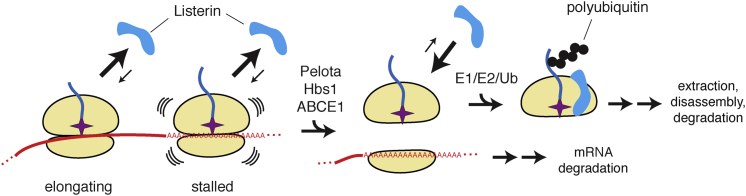
Model for Protein Quality Control Coupled to mRNA Surveillance The absence or skipping of a stop codon results in translation into the poly(A) tail and ribosome stalling. Listerin has poor affinity for either elongating or stalled ribosomes. The latter are selectively targeted by ribosome recycling factors (Pelota, Hbs1, and ABCE1) to generate a 60S-nascent chain complex and 40S-mRNA complex. The 60S-nascent chain complex recruits Listerin to ubiquitinate the nascent chain, while the freed mRNA can be degraded by mRNA surveillance pathways. Additional components of the ribosome quality control complex (RQC; not depicted) may facilitate downstream steps of nascent chain extraction and degradation. Similar events are proposed to occur for stalls at the 3′ end of a message, at internal polybasic domains, or areas of mRNA secondary structure. See also Figure S7.

We have presented a cell-free system that recapitulates nascent polypeptide ubiquitination on stalled RNCs featuring in certain mRNA surveillance pathways. Our mammalian in vitro analysis complements recent in vivo studies in yeast and provides several mechanistic insights into ribosome-associated protein quality control. Prime among these insights is a role for ribosome recycling factors in generating a unique molecular species, the 60S-nascent chain-tRNA complex, that serves as the recognition platform for recruitment of the ubiquitin ligase Listerin (Figure 7). This finding helps order the sequence of events leading to nascent chain ubiquitination and points to RNC recognition by the Hbs1:Pelota complex as an early discriminatory step in this pathway. Given the emerging role for Hbs1:Dom34 in multiple mRNA quality control pathways (Passos et al., 2009; Shoemaker et al., 2010; van den Elzen et al., 2010; Tsuboi et al., 2012), we can further conclude that a commitment to degrade defective mRNAs is mechanistically coupled to degrading the associated polypeptide by virtue of relying on some of the same factors to initiate both pathways.

Ltn1 was previously identified as a ribosome-associated ligase required for nascent chain degradation (Bengtson and Joazeiro, 2010; Brandman et al., 2012). However, the conclusion that Ltn1 directly mediates nascent chain ubiquitination on ribosomes was complicated by the finding that Ltn1 cofractionates with 60S subunits while ΔLtn1 strains accumulate undegraded stalled nascent chains in 80S complexes (Bengtson and Joazeiro, 2010). Our in vitro analysis directly visualized ubiquitinated tRNA-tethered nascent chains (Figure 1C) within the 60S ribosomal subunit (Figure 4A). These complexes contained Listerin, which could be stripped off with high salt to abolish ubiquitination (Figure 3A). Restoration of ubiquitination with cytosol in a Listerin-dependent manner (Figure 3D), together with the genetic data for Ltn1 in yeast, argues strongly that Listerin directly mediates nascent chain ubiquitination at the ribosome.

60S-nascent chain-Listerin complex isolated in a ubiquitination-deficient lysate only required purified E1, E2, ubiquitin, and ATP to trigger nascent chain ubiquitination with high efficiency (Figure 4D). By contrast, 80S-RNCs contained little or no Listerin and were not ubiquitinated under identical conditions (Figure 4D), even when supplemented with Listerin-containing S100 (Figure S7A). Furthermore, ubiquitination reactions in a complete lysate resulted in ubiquitinated substrate almost exclusively in the 60S fractions (Figure 4A). Preventing subunit splitting with Hbs1 depletion or Hbs1-DN reduced nascent chain ubiquitination (Figure 6D). Thus, even when artificially stabilized, stalled 80S RNCs are poor substrates for either Listerin recruitment or ubiquitination. We therefore conclude that 60S RNCs are the site of Listerin-mediated nascent chain ubiquitination.

The simplest model for Listerin recruitment based on the available data is that it binds 60S-nascent chain complexes after ribosome subunit separation. The requirement for both the 60S subunit and the nascent chain (Figure 5), together with the lack of stable binding to 80S RNCs, suggests that the tRNA and/or the subunit interface may be involved in recognition. Both of these features would only become exposed upon subunit separation, providing an explanation for why translating ribosomes are not targeted promiscuously by Listerin. This implies that the decisive and committed step in stalled RNC degradation is subunit dissociation by the ribosome recycling system of Hbs1:Pelota:ABCE1.

Given this conclusion, it was somewhat puzzling that the majority of stalled RNCs in our in vitro assays accumulate as 80S RNCs (e.g., Figure 4A). We initially thought it reflected inefficient ribosome splitting. However, efficient drop-off of a short nascent chain (Figure 5C) and efficient release of radiolabeled transcript from stalled ribosomes (Figure 5A) illustrated that ribosome recycling was occurring properly. We therefore posited that after splitting, free 60S-nascent chain complexes can rapidly reassociate with 40S subunits before the samples are analyzed. Indeed, in vitro ribosome splitting assays cannot detect subunit dissociation unless rapid reassociation is prevented by excess eIF6 (e.g., Pisareva et al., 2011). Using the same strategy in our in vitro system, we could illustrate that preventing subunit reassociation resulted in essentially all of the truncated nascent chains in 60S complexes (Figure S7B). Thus, ribosomes reaching the end of a truncated mRNA are efficiently split but appear to reassociate rapidly during sample analysis.

Given that Listerin-containing complexes are preferentially 60S, it appears that binding of Listerin (or perhaps another RQC component) prevents reassociation of the 40S by occluding a portion of the intersubunit interface. Such a model would explain why Listerin has high specificity for 60S and not 80S, why only a small amount of our RNCs are found as 60S complexes despite efficient splitting activity, and why a very high proportion of our 60S complexes contain Listerin. Rapid subunit reassociation in the absence of Listerin-mediated trapping may also explain why undegraded RNCs in ΔLtn1 yeast are found in 80S fractions (Bengtson and Joazeiro, 2010). Similarly, limiting amounts of Ltn1 combined with subunit reassociation may explain why undegraded nascent chains in Cdc48 mutant cells are primarily in 80S RNCs (Verma et al., 2013; Defenouillère et al., 2013).

While it is clear that 80S RNCs bind to Listerin very poorly relative to 60S-nascent chain complexes, we cannot entirely exclude Listerin recruitment initially to the former. In this view, Listerin recruitment would be followed promptly and efficiently by ribosome splitting, explaining why little or no 80S RNCs containing Listerin are observed. Future studies are needed to examine whether Listerin might stimulate ribosome splitting, although an obligate role would seem unlikely based on earlier functional studies in vitro (Pisareva et al., 2011; Shoemaker and Green, 2011).

At present, the upstream steps preceding Listerin recruitment to ribosomes are poorly understood. In yeast, stalling at a polybasic stretch requires both Hel2 and Asc1 (Kuroha et al., 2010; Brandman et al., 2012). How these factors prevent translation elongation at polybasic domains or how this step is related to subsequent ribosome splitting is not clear. Similarly, it remains to be determined whether these factors play any role in resolving stalls that are at the end of a message, or that are caused by physical barriers such as RNA secondary structure. Regardless of this uncertainty, the next step in each case is likely to be ribosome dissociation. How the Hbs1:Pelota complex distinguishes between stalled, elongating, and terminating RNCs is not clear.

Finally, the steps downstream of 60S-nascent chain ubiquitination remain to be elucidated. This presumably involves nascent chain extraction from the 60S subunit followed by delivery to the proteasome. The Cdc48 complex together with its adaptors are found on Ltn1-containing 60S complexes (Brandman et al., 2012; Verma et al., 2013; Defenouillère et al., 2013), suggesting a possible role in extracting the nascent chain. Indeed, Cdc48 depletion results in accumulation of undegraded nascent chains on the ribosome (Verma et al., 2013; Defenouillère et al., 2013). Our in vitro system, and the ability to prepare 60S-nascent chain-Listerin complexes, should now permit these downstream steps to be analyzed in mechanistic detail.

## Experimental Procedures

### Plasmids and Antibodies

The SP64 vector-based constructs encoding PrP, GFP, and Sec61β-ΔTMD have been described (Kang et al., 2006; Stefanovic and Hegde, 2007; Sharma et al., 2010; Hessa et al., 2011). The VHP domain (McKnight et al., 1996) was inserted between amino acids 14 and 15 of Sec61β-ΔTMD. The K12 domain (encoded by 12 AAA codons) and stem loop (Doma and Parker, 2006) were inserted just before the C-terminal tag. For affinity purification, a 3× HA tag was inserted at the N terminus. Pelota cDNA was cloned into the pRSETA vector for bacterial expression. Hbs1 cDNA was cloned into a pcDNA3.1-based vector containing an N-terminal 3X-FLAG tag. GTPase mutants (V269G and H348A) were made by mutagenesis. The Anti-Ltn1 (Abcam), anti-Hbs1 (Abcam), anti-RPL9 (Santa Cruz), and anti-RPS16 (Santa Cruz) antibodies were purchased. The anti-HA, anti-Sec61β, and anti-3F4 epitope antibodies were raised in-house against KLH-conjugated peptides. Anti-HA and anti-FLAG affinity resins were from Sigma.

### Transcription and Translation

All transcripts utilized PCR products as templates for in vitro transcription (Sharma et al., 2010) except β-VHP-SL, which was transcribed from linearized plasmid. β-VHP-pA transcript was generated by treating β-VHP transcript with poly(A) polymerase to add an ∼200 nt poly(A) tail. ^33^P-labeled transcripts were generated with ^33^P-UTP (Perkin-Elmer) in transcription reactions. In vitro translation reactions using rabbit reticulocyte lysate (RRL), phenyl-depleted RRL, and DEAE fractionated RRL (Fr-RRL) were done as before (Sharma et al., 2010; Hessa et al., 2011). ΔHbs1 RRL was generated by incubating 800 μl RRL with 200 μl of Pelota resin (immobilized via CnBr). Unconjugated and quenched CnBr resin served as a control. Unless indicated otherwise, translation reactions were for 60 min at 32°C. Where indicated, WT or DN Hbs1 was added at 10 min together with 100 μM aurin tricarboxylic acid to inhibit initiation. For direct analyses, translation reactions were denatured in 1% SDS and heated to 100°C. For downstream applications, translation reactions were cooled on ice and manipulated at 0°C–4°C for RNC isolation, sucrose gradients, and native immunoprecipitations (IPs).

### Sucrose Gradients and RNC Preparation

Gradients were either 2 ml 10%–50% sucrose or 4.8 ml 10%–30% sucrose, both in RNC buffer (RB, 50 mM HEPES [pH 7.4], 100 mM KAc, 5 mM MgCl_2_). Centrifugation was at 55,000 rpm (TLS-55 rotor) for 1 hr or 50,000 rpm (MLS-50 rotor) for 2 or 2.5 hr at 4°C. Fractions (200 μl) were collected from the top. Native (“low salt”) and high salt-stripped RNCs were isolated from translation reactions by sedimentation through a 1.6 ml 0.5 M sucrose cushion in either RB or high salt buffer (50 mM HEPES [pH 7.4], 750 mM KAc, 15 mM MgCl_2_). The RNC pellets were resuspended in RB+10% sucrose and used immediately or flash frozen in liquid nitrogen.

### Ubiquitination Assays

Ubiquitination during translation used 10 μM His-tagged or FLAG-tagged ubiquitin (Boston Biochem). Posttranslational ubiquitination of RNCs contained 75 nM E1, 250 nM UbcH5a, an energy regenerating system (ERS, 1 mM ATP, 1 mM GTP, 12 mM creatine phosphate, 20 μg/ml creatine kinase), and 10 μM His-ubiquitin or FLAG-ubiquitin. Reactions were incubated at 32°C for 45 min, stopped by the addition of 1% SDS, and boiled.

### Cell Culture

HEK293T cells were cultured in DMEM with 10% FBS. Cells were harvested when ∼50%–70% confluent in lysis buffer (25 mM HEPES, 125 mM KAc, 15 mM MgAc_2_, 40U/mL RNasin, 100 μg/mL digitonin, 50 μg/mL cycloheximide, 1× protease inhibitor cocktail, 1 mM DTT). Where indicated, cells were pretreated with either 200 nM pactamycin or 50 μg/mL cycloheximide for 1 hr prior to harvest. After removing debris and nuclei by centrifugation, the lysate was analyzed by sucrose gradients and immunoblotting as described above.

### Miscellaneous Biochemistry

S-100 was generated by removing ribosomes from RRL by centrifugation. Listerin was immunodepleted from S-100 by two rounds of incubation with 50 μg of protein A-bound anti-Listerin or control antibody. His-tagged Pelota was expressed and purified from BL21(DE3) pLysS *E. coli.* Flag-tagged Hbs1 proteins were expressed and purified from HEK293T cells (Hessa et al., 2011). Denaturing pull-downs of His-tagged ubiquitinated products was with Ni^2+^ or Co^2+^ immobilized on chelating Sepharose (Hessa et al., 2011). Immunoprecipitations and CTAB precipitations were as before (Mariappan et al., 2010). SDS-PAGE was on 8% or 12% tricine gels.

## Supplemental Information

Document S1. Figure S1, Figure S2, Figure S3, Figure S4, Figure S5, Figure S6, Figure S7, and Supplemental Experimental Procedures
