# Supplementary material for: Listerin-Dependent Nascent Protein Ubiquitination Relies on Ribosome Subunit Dissociation
Source: Mol Cell. 2013 Jun 6;50(5):637–48. doi: 10.1016/j.molcel.2013.04.015 (PMC3719020; doi:10.1016/j.molcel.2013.04.015)
Supplement: Document S1. Figure S1, Figure S2, Figure S3, Figure S4, Figure S5, Figure S6, Figure S7, and Supplemental Experimental Procedures [file mmc1.pdf]

Molecular Cell, Volume *50*

Supplemental Information

Listerin-Dependent Nascent Protein Ubiquitination Relies on Ribosome Subunit Dissociation

Sichen Shao, Karina von der Malsburg, and Ramanujan S. Hegde

SUPPLEMENTAL INFORMATION INVENTORY

Supplemental Data

Sup. Fig. S1 (related to Fig. 1). Detection of nascent chain ubiquitination on the ribosome.

Sup Fig. S2 (related to Fig. 2). Ubiquitination of stalled tRNA-associated nascent chains.

Sup Fig. S3 (related to Fig. 3). Characteristics of ribosome-associated nascent chain ubiquitination.

Sup. Fig. S4 (related to Fig. 4). 60S-nascent chain complexes are preferentially ubiquitinated.

Sup. Fig. S5 (related to Fig. 5). Listerin does not recognize free tRNA-tethered nascent chains.

Sup. Fig. S6 (related to Fig. 6). Hbs1-mediated ribosome splitting precedes ubiquitination.

Sup. Fig. S7 (related to Fig. 7). 80S RNCs are not efficient substrates for ubiquitination.

Supplemental Experimental Procedures

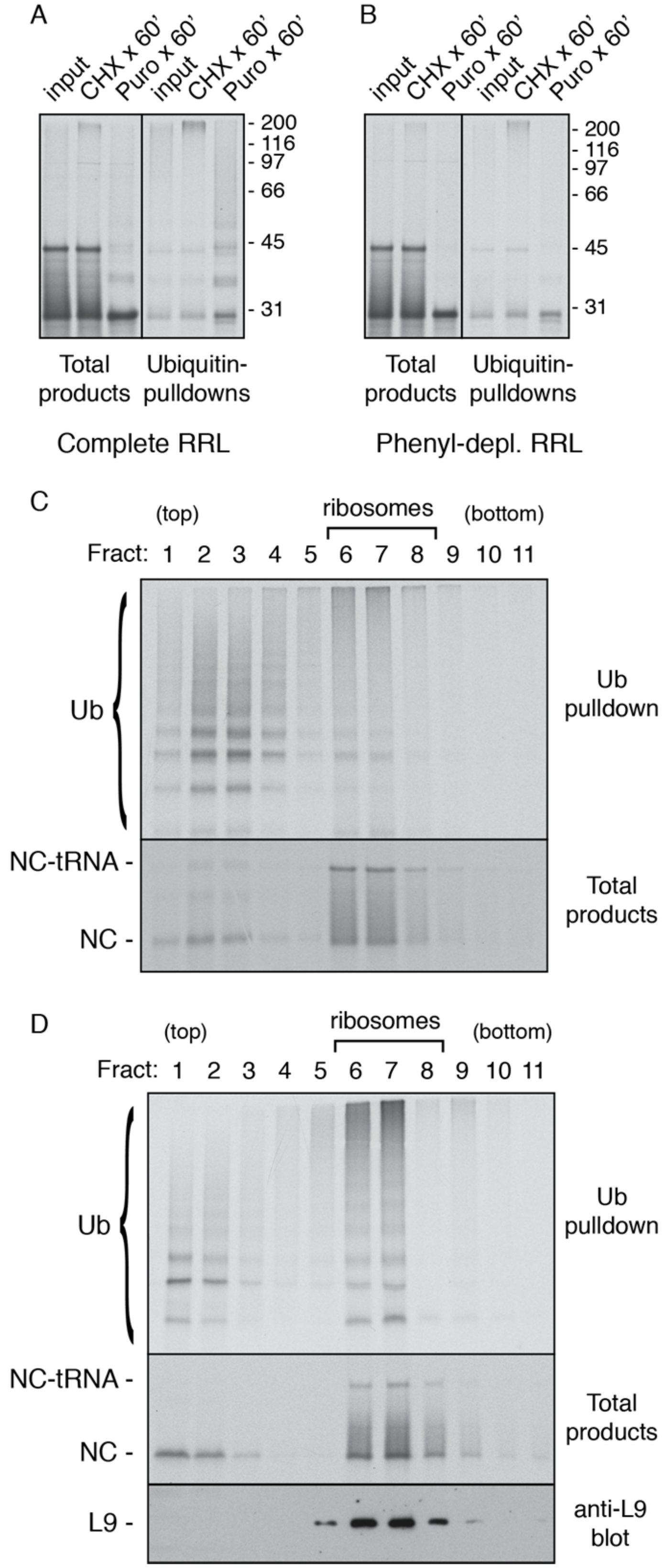
A
input
CHX x 60'
Puro x 60'
input
CHX x 60'
Puro x 60'
- 200
- 116
- 97
- 66
- 45
- 31
Total products
Ubiquitin-pulldowns
Complete RRL
B
input
CHX x 60'
Puro x 60'
input
CHX x 60'
Puro x 60'
- 200
- 116
- 97
- 66
- 45
- 31
Total products
Ubiquitin-pulldowns
Phenyl-depl. RRL
C
(top)
ribosomes
(bottom)
Fract: 1 2 3 4 5 6 7 8 9 10 11
Ub
Ub pulldown
NC-tRNA -
NC -
Total products
D
(top)
ribosomes
(bottom)
Fract: 1 2 3 4 5 6 7 8 9 10 11
Ub
Ub pulldown
NC-tRNA -
NC -
Total products
L9 -
anti-L9 blot

Sup. Fig. S1 (related to Fig. 1). Detection of nascent chain ubiquitination on the ribosome. (A) RNCs encoding a modified prion protein (PrP) were produced by in vitro translation in RRL for 30 min. The translation reaction contained 10 μM His-Ubiquitin. An aliquot of the initial translation reaction was reserved for direct analysis (input) and the remainder was divided for incubation with 100 μg/ml Cycloheximide (CHX) or 1 mM Puromycin (Puro) for 1 hr at 32°C. The samples were denatured by boiling in 1% SDS and analyzed directly (Total products) or subjected to immobilized Co^{2+} pulldowns to enrich for ubiquitinated species (Ubiquitin pulldowns). The positions of the nascent-chain-tRNA (NC-tRNA) and nascent chain (NC) are indicated. Note that upon puromycin treatment, the translation product is released from the ribosome as evidenced by loss of the tRNA. The released product is ubiquitinated as illustrated by the ladder/smear observed in the ubiquitin pulldowns. The CHX sample shows a high molecular weight smear at the top of the gel that also proved to be ubiquitinated products as evidenced by its pulldown by immobilized Co^{2+}. **(B)** An experiment identical to panel A was performed in a phenyl-depleted RRL translation extract. This lysate lacks the cytosolic quality control factors that normally ubiquitinate PrP in the cytosol, and hence PrP ubiquitination upon release with puromycin is markedly reduced relative to panel A. By contrast, the high molecular weight smear in the CHX sample is unaffected. This suggests that nascent PrP on the ribosome is ubiquitinated by a pathway that is different than that used for PrP released into the cytosol. It is noteworthy that ubiquitination on RNCs appears to be more processive than ubiquitination after release into the cytosol. **(C, D)** RNCs of PrP (panel C) and GFP (panel D) were produced in RRL containing His-tagged ubiquitin and separated on a 10-50% sucrose gradient. Each fraction was analyzed directly (Total products) or enriched for ubiquitinated products with immobilized Co^{2+} (Ub pulldown). An aliquot of each fraction was also immunoblotted with an antibody against ribosomal protein L9 to detect the position of ribosomes. The positions of the nascent chain (NC) and NC-tRNA are indicated. Note that tRNA association is only seen in the ribosome fractions (primarily 6 and 7). Ubiquitination is observed in these fractions for both substrates. The tRNA is partially hydrolyzed during sample preparation and electrophoresis, resulting in a smear between the NC-tRNA and NC bands. Analysis of various substrates of different length and sequence by similar methods (data not shown) illustrated that ubiquitination on RNCs was substrate-independent.

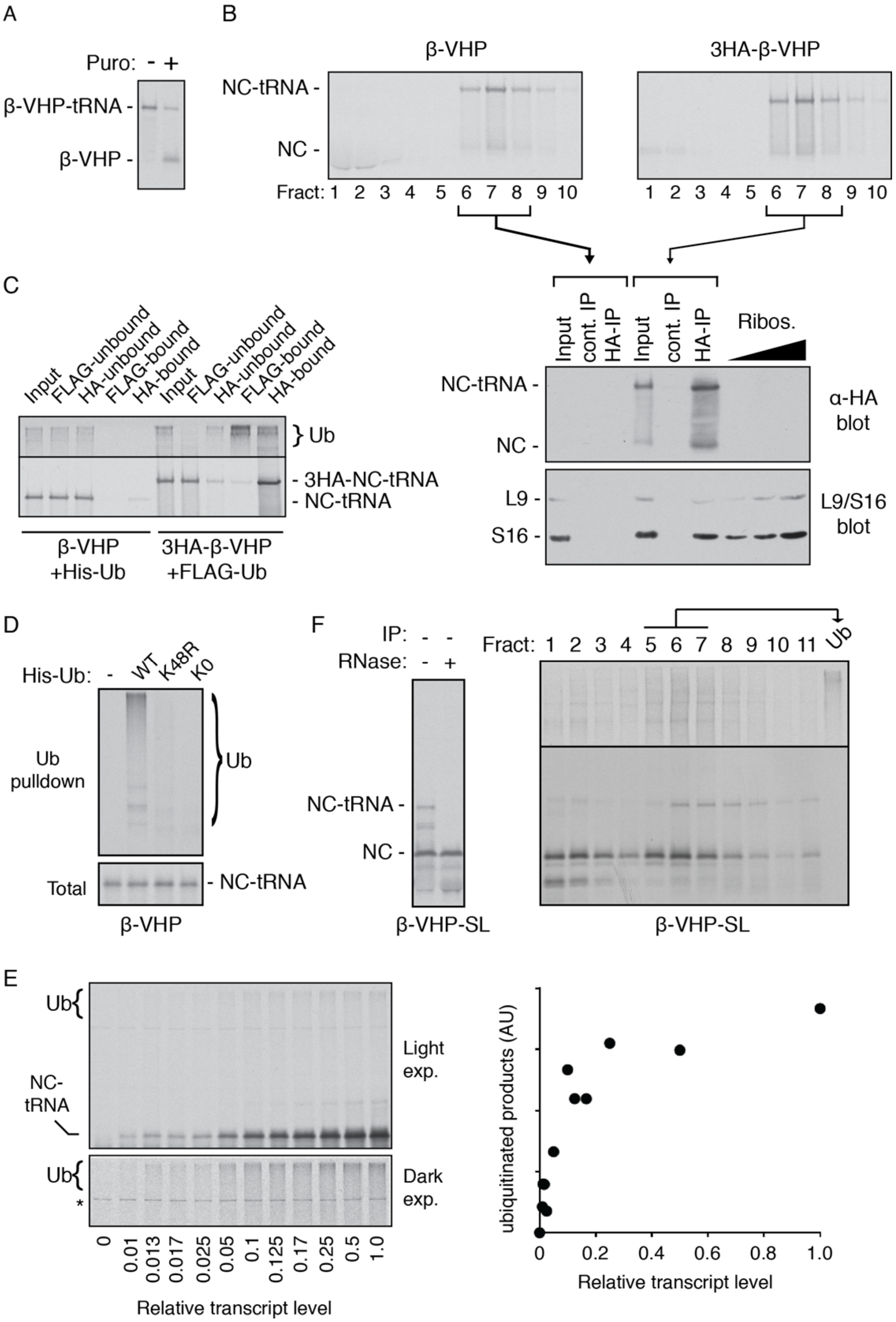
A
B
Puro: - +
β-VHP-tRNA -
β-VHP -
β-VHP
3HA-β-VHP
NC-tRNA -
NC -
Fract: 1 2 3 4 5 6 7 8 9 10
C
Input
FLAG-unbound
HA-unbound
FLAG-bound
HA-bound
} Ub
- 3HA-NC-tRNA
- NC-tRNA
β-VHP +His-Ub
3HA-β-VHP +FLAG-Ub
Input
cont. IP
HA-IP
Ribos.
α-HA blot
L9 -
S16 -
L9/S16 blot
D
His-Ub: - WT K48R K0
Ub pulldown
Ub
Total
β-VHP
F
IP: - -
RNase: - +
Fract: 1 2 3 4 5 6 7 8 9 10 11 Ub
β-VHP-SL
E
Ub
NC-tRNA
Light exp.
Dark exp.
*
0 0.01 0.013 0.017 0.025 0.05 0.1 0.125 0.17 0.25 0.5 1.0
Relative transcript level
ubiquitinated products (AU)
0 0.2 0.4 0.6 0.8 1.0

Sup Fig. S2 (related to Fig. 2). Ubiquitination of stalled tRNA-associated nascent chains. (A) β-VHP RNCs were produced in RRL and separated on a 10-50% sucrose gradient. The pooled RNC fractions (6-8; see Fig. 1D) was incubated with or without puromycin (Puro) and the products analyzed by SDS-PAGE. The addition of puromycin releases β-VHP from the P-site tRNA (β-VHP-tRNA), resulting in a decrease in the size of the product. **(B)** β-VHP or 3X HA-tagged β-VHP (3HA-β-VHP) RNCs were translated and separated on a 10-50% sucrose gradient, and the individual fractions analyzed by SDS-PAGE (top panels). Ribosomal fractions containing the tRNA-associated nascent chains (NC-tRNA) were pooled and subject to native immunoprecipitations with control or anti-HA antibodies as indicated. The IPs were analyzed together with 20% input and a titration of purified ribosomes by immunoblotting for HA-tagged substrate (top) and for the ribosomal proteins L9 and S16 (bottom). We estimate that based on this blot (and adjusting for ~80% recovery in the IPs), around 10-15% of the total ribosomes in the translation reaction were programmed with the 3HA-β-VHP VHP nascent chains. **(C)** β-VHP and 3X HA-tagged β-VHP RNCs were translated in RRL containing His-tagged ubiquitin and FLAG-tagged ubiquitin, respectively. The reactions were separated on a 10-50% sucrose gradient, the RNC fractions were pooled and subjected to denaturing immunoprecipitation with anti-FLAG and anti-HA resins. Equivalent amounts of the input and unbound material were analyzed along with 4-fold excess of the immunoprecipitated sample. A short exposure (bottom) and a long exposure (top) of the lower and upper portions of the gel are displayed to illustrate recovery of the substrate and ubiquitinated products, respectively. **(D)** β-VHP RNC was translated in the absence or presence of His-tagged wild type, K48R, or K0 ubiquitin. The total amount of translated β-VHP-tRNA (bottom) and purified ubiquitinated products (top) are shown. K48R and K0 ubiquitin lack the ability to generate polyubiquitin chains on the substrate. **(E)** Different relative amounts of β-VHP transcript were used to program translation reactions, and the total products were analyzed by SDS-PAGE. A darker exposure of the top part of the gel is shown in the lower panel to better visualize the ubiquitinated products. The amount of ubiquitinated products in each lane was quantified and plotted in the graph. Ubiquitination nears saturation at a relative transcript level of ~0.1. Above this point, little or no increase in ubiquitination is seen despite increasing substrate synthesis. **(F)** Full-length β-VHP containing a stem-loop before a C-terminal tag was translated and subject to RNase treatment (left), or separated on a 10-50% gradient (right). The individual fractions were immunoprecipitated with an antibody against the substrate, and the ribosomal fractions pooled and subject to pulldowns against tagged ubiquitin. Note that stalling is not complete, but tRNA-associated nascent chain (NC-tRNA) migrates in ribosomal fractions, which contain ubiquitinated substrate.

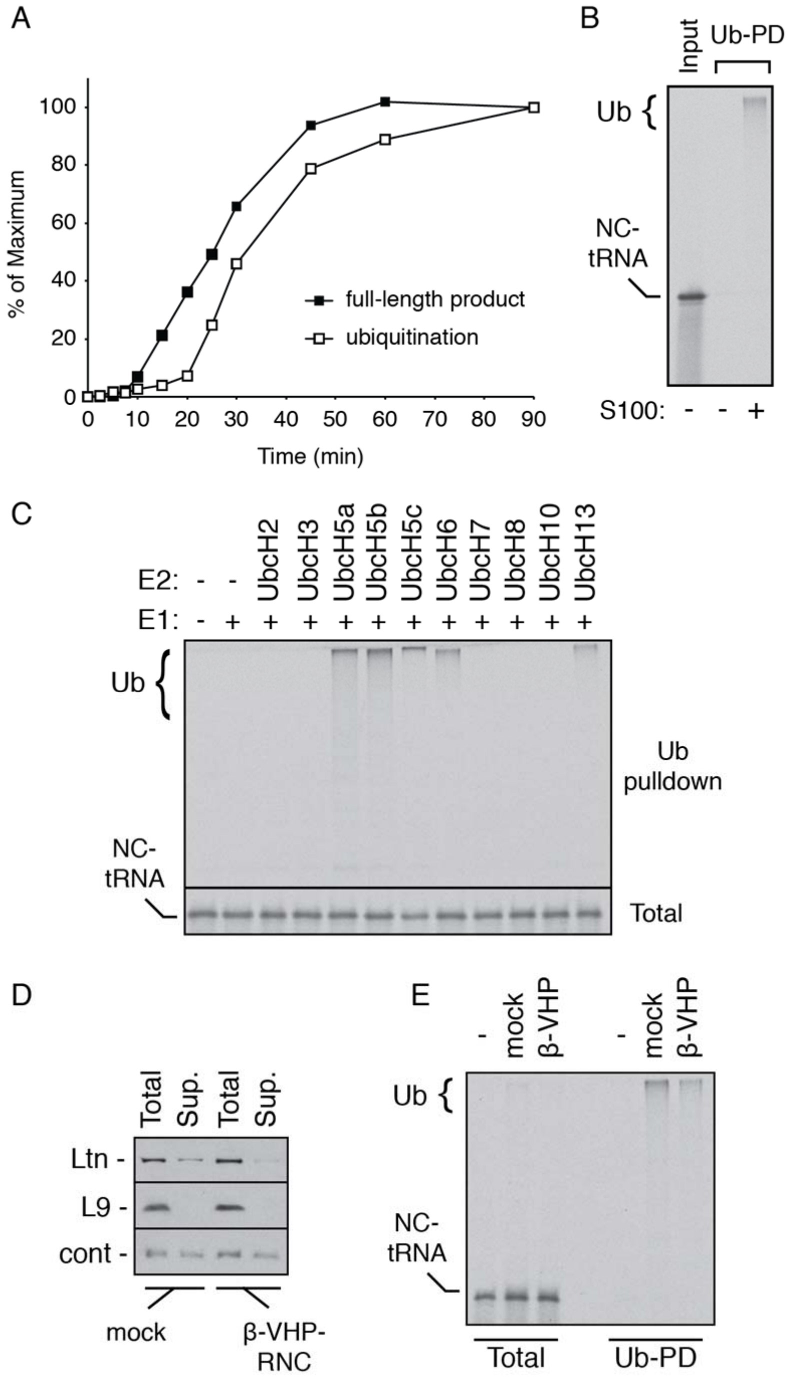
A
% of Maximum
Time (min)
full-length product
ubiquitination
B
Input
Ub-PD
Ub
NC-tRNA
S100: - - +
C
E2: - - UbcH2 UbcH3 UbcH5a UbcH5b UbcH5c UbcH6 UbcH7 UbcH8 UbcH10 UbcH13
E1: - + + + + + + + + + + +
Ub
NC-tRNA
Ub pulldown
Total
D
Total Sup. Total Sup.
Ltn -
L9 -
cont -
mock
β-VHP-RNC
E
- mock β-VHP - mock β-VHP
Ub
NC-tRNA
Total
Ub-PD

Sup Fig. S3 (related to Fig. 3). Characteristics of ribosome-associated nascent chain ubiquitination. (A) β-VHP RNCs were translated in the presence of His-tagged ubiquitin for the indicated time points before rapid denaturation with boiling SDS. An aliquot was saved for direct analysis of the full-length product while the remainder was subject to His pulldowns to isolate ubiquitinated products. All samples were analyzed by SDS-PAGE, exposed to a phosphorimager, and quantified using a Typhoon scanner. The amount of substrate and ubiquitinated products at each time point relative to 90 min is plotted. **(B)** β-VHP was translated and the RNCs isolated by centrifugation under physiological salt conditions (input). The isolated RNCs (input) were incubated with or without S100 cytosol in the presence of His-tagged ubiquitin and energy. Purified ubiquitinated products are shown, revealing that the S100 cytosol contains sufficient machinery for RNC ubiquitination. **(C)** β-VHP was translated and the RNCs isolated by centrifugation under physiological salt conditions. The isolated RNCs were incubated with various E2 enzymes (at 100 nM each) in the presence of 75 nM E1 enzyme, His-tagged ubiquitin, and energy. Total products of the reaction and purified ubiquitinated products are displayed in the bottom and top panels, respectively. The Ubc4/5 family members (UbcH5a, b, c, and UbcH6) are the preferred E2 enzymes for this substrate, while UbcH13 shows lower activity. The others did not show any activity. **(D)** Non-radioactive translation reactions lacking (mock) or containing truncated β-VHP transcript were analyzed directly (Total) or after ribosomes were removed by centrifugation (S100). Shown are blots for Listerin, the ribosomal protein L9, and an unrelated control protein. Note that translation of β-VHP RNC depletes more Listerin from the S100 than the mock translation. **(E)** The S100 fractions from panel D were incubated with high salt-stripped β-VHP RNCs in the presence of His-tagged ubiquitin, E1 and E2 enzymes, and energy. The reactions were analyzed directly (Total) or subject to His pulldowns (Ub-PD). The S100 from the β-VHP RNC translation shows ~30-40% activity relative to the S100 from the mock translation.

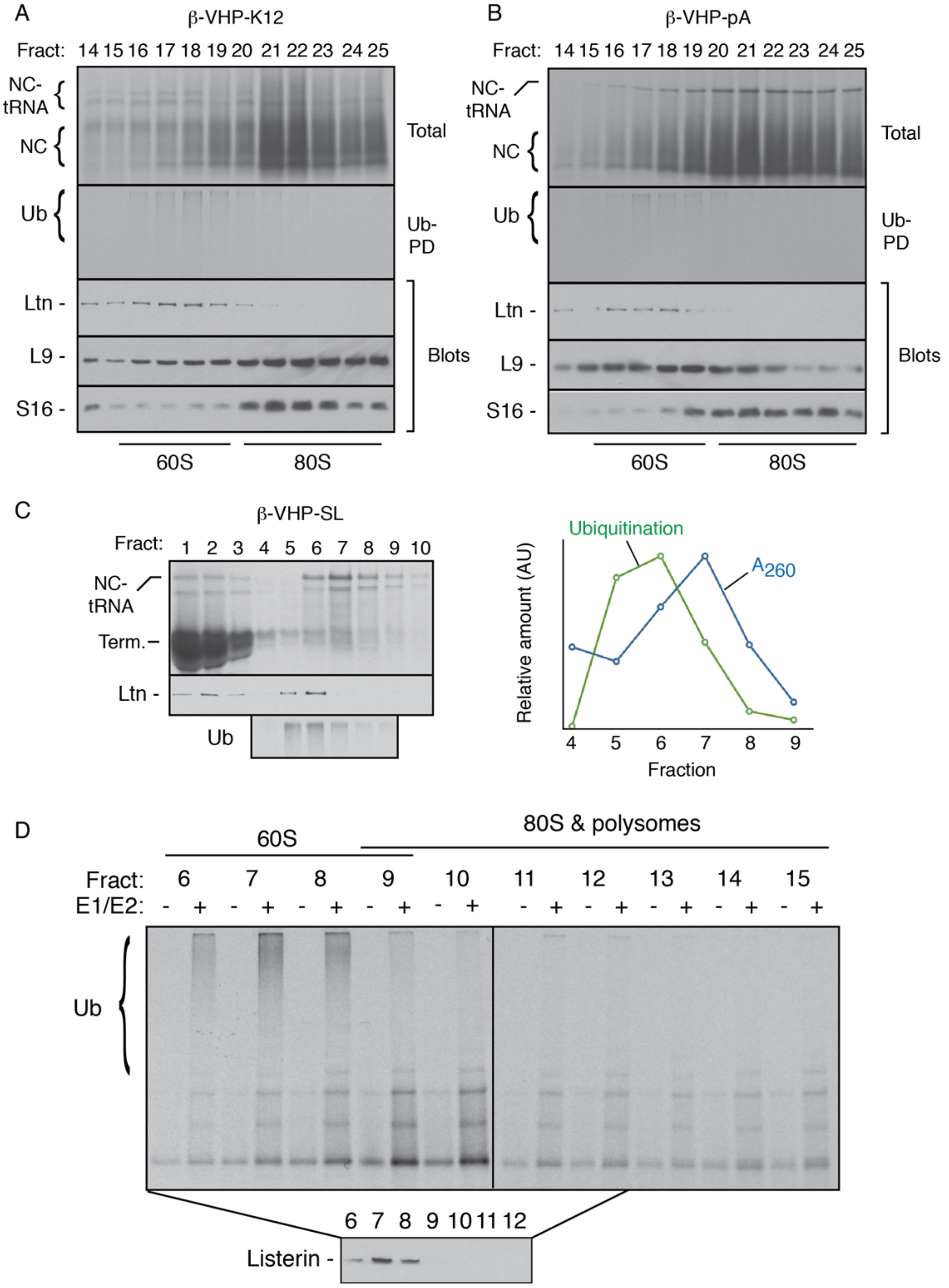
A
β-VHP-K12
Fract: 14 15 16 17 18 19 20 21 22 23 24 25
NC-tRNA
NC
Total
Ub
Ub-PD
Ltn -
L9 -
S16 -
Blots
60S
80S
B
β-VHP-pA
Fract: 14 15 16 17 18 19 20 21 22 23 24 25
NC-tRNA
NC
Total
Ub
Ub-PD
Ltn -
L9 -
S16 -
Blots
60S
80S
C
β-VHP-SL
Fract: 1 2 3 4 5 6 7 8 9 10
NC-tRNA
Term.
Ltn -
Ub
Ubiquitination
A260
Relative amount (AU)
4 5 6 7 8 9
Fraction
D
60S
80S & polysomes
Fract: 6 7 8 9 10 11 12 13 14 15
E1/E2: - + - + - + - + - + - + - + - + - + - +
Ub
6 7 8 9 10 11 12
Listerin -

Sup. Fig. S4 (related to Fig. 4). 60S-nascent chain complexes are preferentially ubiquitinated. (A, B) β-VHP-K12 and β-VHP-pA RNCs were produced in the presence of His-tagged ubiquitin and separated on a 10-30% sucrose gradient. Individual fractions were analyzed for ubiquitinated products via His pulldown (Ub-PD), the nascent chain (total), Listerin, and ribosomal proteins of the 60S (L9) and 40S (S16) subunits. Fractions with 60S, and 80S complexes are shown. For both constructs, Listerin and the ubiquitinated nascent chains co-sediment with 60S subunits. **(C)** β-VHP-SL was translated in the presence of His-tagged ubiquitin and separated on a 10-50% sucrose gradient. Individual fractions were analyzed directly (top panel), for Listerin (middle panel) or for ubiquitinated products via His-pulldown (bottom panel). Note that a substantial amount of ribosomes read through the stem-loop and terminate to give a complete protein ('Term.'), while a relative minority stall at the stem-loop and generate a nascent chain-tRNA product. The graph shows the relative amount of ubiquitinated products and A_{260} readings in fractions 4-9. Note that while the peak A_{260} reading (corresponding to 80S ribosomes) is in fraction 7, the majority of Listerin and ubiquitination is seen in fractions 5 and 6, corresponding to 60S subunits. **(D)** PrP RNCs translated for 30 min (before substantial ubiquitination had occurred) were separated on a high-resolution sucrose gradient and the fractions corresponding to 60S and 80S (as judged by A260 profiles) collected. Each fraction was divided in two and incubated without or with E1 and E2 enzymes plus His-tagged ubiquitin and an ATP regenerating system. The ubiquitinated products were enriched by pulldown using immobilized Co^{2+} and analyzed by autoradiography (top panel). Aliquots of the sucrose gradient fractions were also analyzed by immunoblotting for Listerin (bottom panel). The 60S and 80S fractions are marked. Note that the 60S-nascent chain-tRNA products are preferentially polyubiquitinated, and that this ubiquitination activity co-fractionates with Listerin.

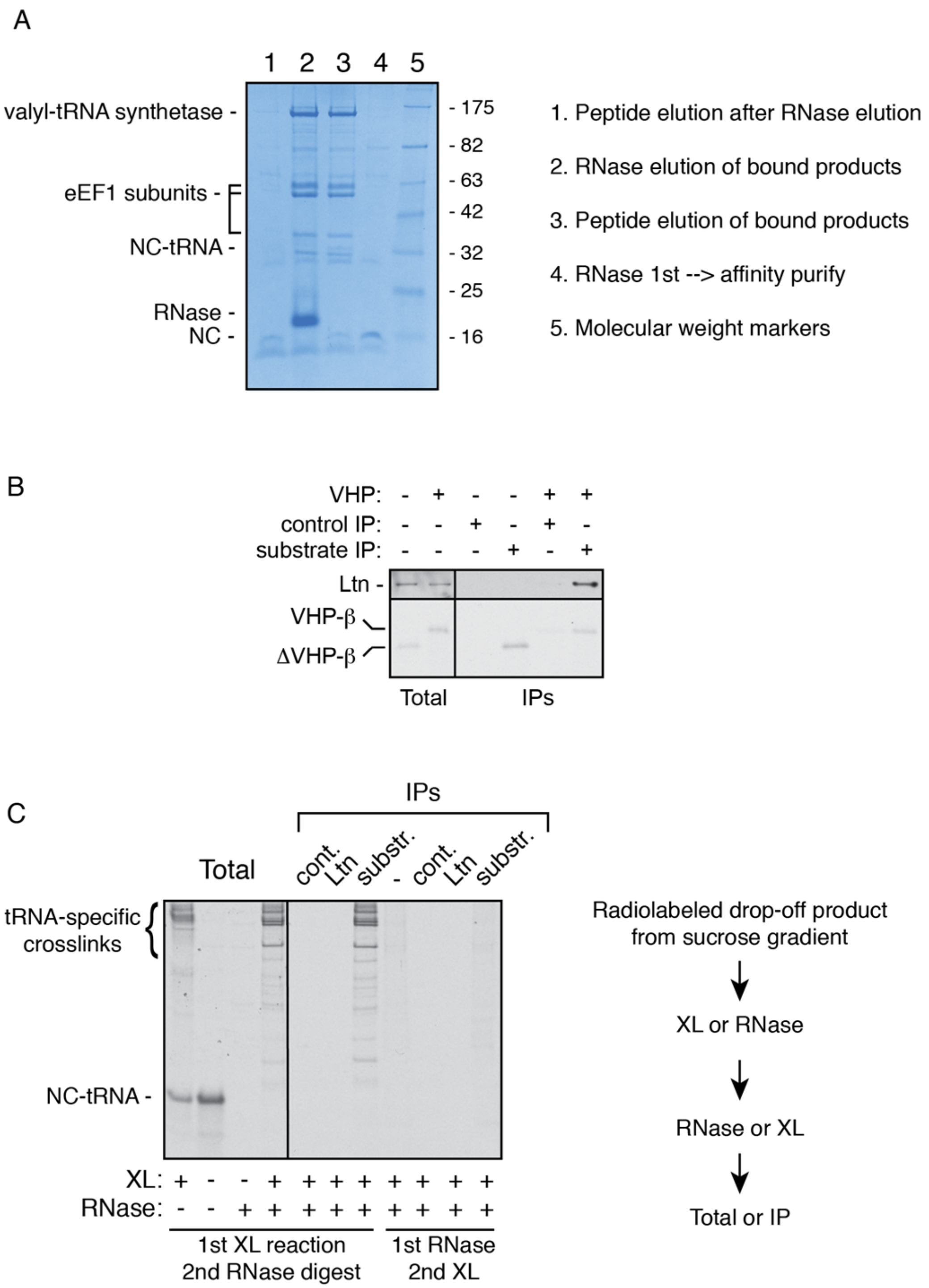
A
1 2 3 4 5
valyl-tRNA synthetase -
eEF1 subunits -
NC-tRNA -
RNase -
NC -
- 175
- 82
- 63
- 42
- 32
- 25
- 16
1. Peptide elution after RNase elution
2. RNase elution of bound products
3. Peptide elution of bound products
4. RNase 1st --> affinity purify
5. Molecular weight markers
B
VHP: - + - - + +
control IP: - - + - + -
substrate IP: - - - + - +
Ltn -
VHP-β
ΔVHP-β
Total
IPs
C
IPs
Total
cont.
Ltn
substr.
-
cont.
Ltn
substr.
tRNA-specific crosslinks
NC-tRNA -
XL: + - - + + + + + + + +
RNase: - - + + + + + + + + +
1st XL reaction
2nd RNase digest
1st RNase
2nd XL
Radiolabeled drop-off product from sucrose gradient
XL or RNase
RNase or XL
Total or IP

Sup. Fig. S5 (related to Fig. 5). Listerin does not recognize free tRNA-tethered nascent chains. (A) 3X-Flag tagged β RNCs were translated to generate free nascent chain-tRNA (NC-tRNA) that had slipped out of split ribosomes as in Fig. 5C. Ribosomes were spun down and the supernatant was directly incubated with anti-Flag resin (lanes 1-3) or treated with RNase (lane 4) before being bound to anti-Flag resin. The resin was washed and eluted with RNase treatment (lane 2) followed by a Flag-peptide elution (lane 1) or subjected directly to a peptide elution (lanes 3 and 4). The lanes were subject to mass spectrometry, which identified the primary tRNA-specific interactors as valyl synthetase and components of the eEF1 complex. **(B)** 3X-Flag tagged β or VHP-β RNCs were translated to generate free NC-tRNA or ribosome-associated NC-tRNA, respectively (see Fig. 5C). The two samples were analyzed directly (Total) or subject to immunoprecipitations under native conditions with anti-Flag (against the substrate) or anti-HA (control). Blotting for Listerin reveals that it preferentially associates with the ribosome-associated VHP-β RNCs, but not the released β NC-tRNA. **(C)** Radiolabeled β RNC was translated and separated on a 5-25% gradient. Peak fractions containing tRNA-specific interactors (as judged by preliminary crosslinking experiments) were pooled and subject to BMH crosslinking and/or RNase treatment in the indicated order. Note the tRNA-specific crosslinks that are present only if crosslinking is conducted before RNase treatment. Crosslinked samples were then subject to immunoprecipitations with non-immune serum (control), or antibodies against Listerin or the N-terminus of the substrate. Similar results were obtained with amine-reactive crosslinker (not shown).

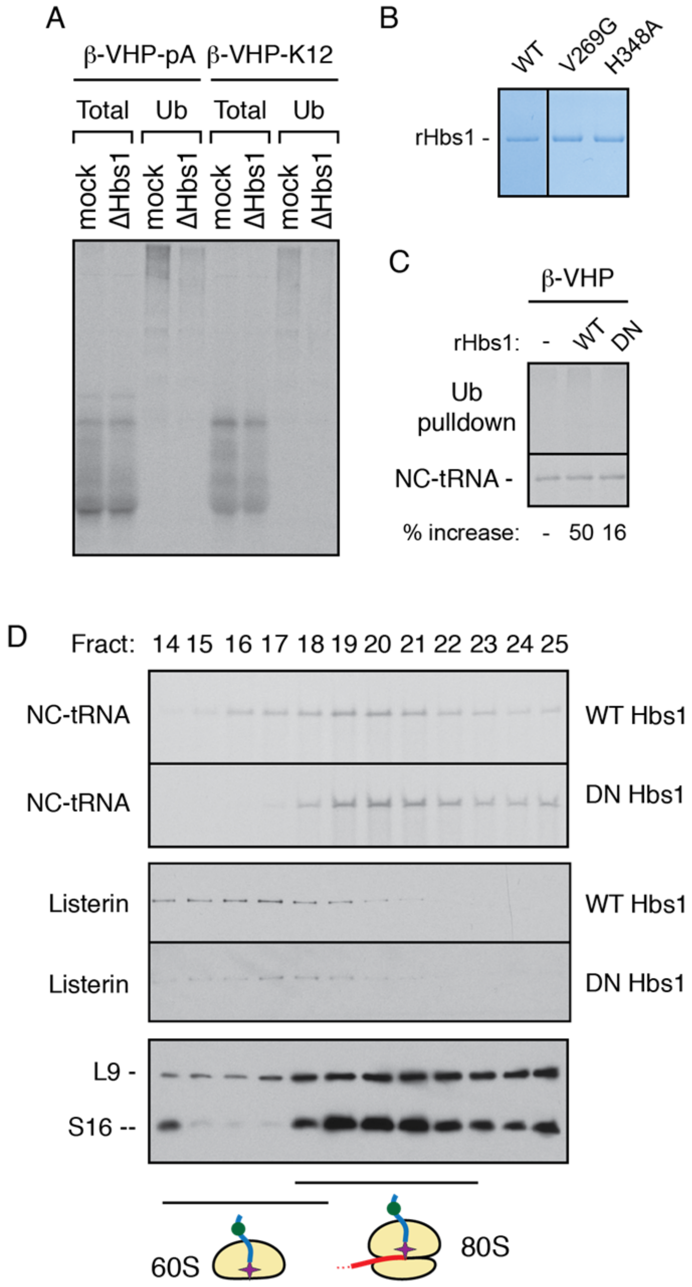
A
β-VHP-pA
β-VHP-K12
Total
Ub
Total
Ub
mock
ΔHbs1
mock
ΔHbs1
mock
ΔHbs1
mock
ΔHbs1
B
WT
V269G
H348A
rHbs1 -
C
β-VHP
rHbs1:
-
WT
DN
Ub pulldown
NC-tRNA -
% increase:
-
50
16
D
Fract: 14 15 16 17 18 19 20 21 22 23 24 25
NC-tRNA
WT Hbs1
NC-tRNA
DN Hbs1
Listerin
WT Hbs1
Listerin
DN Hbs1
L9 -
S16 --
60S
80S

Sup. Fig. S6 (related to Fig. 6). Hbs1-mediated ribosome splitting precedes ubiquitination. (A) β-VHP-pA and β-VHP-K12 were translated in mock or Hbs1-depleted (ΔHbs1) RRL in the presence of His-tagged ubiquitin, and analyzed directly by SDS-PAGE or subject to pulldowns of ubiquitinated species. Both substrates show reduced ubiquitination in Hbs1-depleted reactions. **(B)** Coomassie stain of WT and two GTPase-deficient mutants of tagged Hbs1 purified from HEK 293T cells. **(C)** β-VHP was translated in ΔHbs1 RRL in the absence or presence of WT Hbs1 or H348A mutant Hbs1 (DN) and subject to pulldowns against tagged ubiquitin. Quantification of the change in ubiquitination is included below each lane. Note that WT, but not DN, Hbs1, can partially restore ubiquitination levels in ΔHbs1 lysate. **(D)** β-VHP RNCs were translated with an excess of either WT or DN Hbs1 and separated on a high-resolution 10-30% sucrose gradient. Fractions containing 60S and 80S ribosomes were analyzed for substrate (NC-tRNA) and blotted for Listerin content and ribosomal proteins (L9 and S16). DN Hbs1 impedes splitting of stalled ribosomes, as evidenced by the reduction of substrate in 60S fractions. Of note, the total amount of 60S and 80S ribosomes in the lysate was not visibly affected by DN Hbs1. Listerin recruitment to ribosomal fractions is reduced in the presence of DN Hbs1, and what is recruited predominantly co-fractionates with 60S subunits.

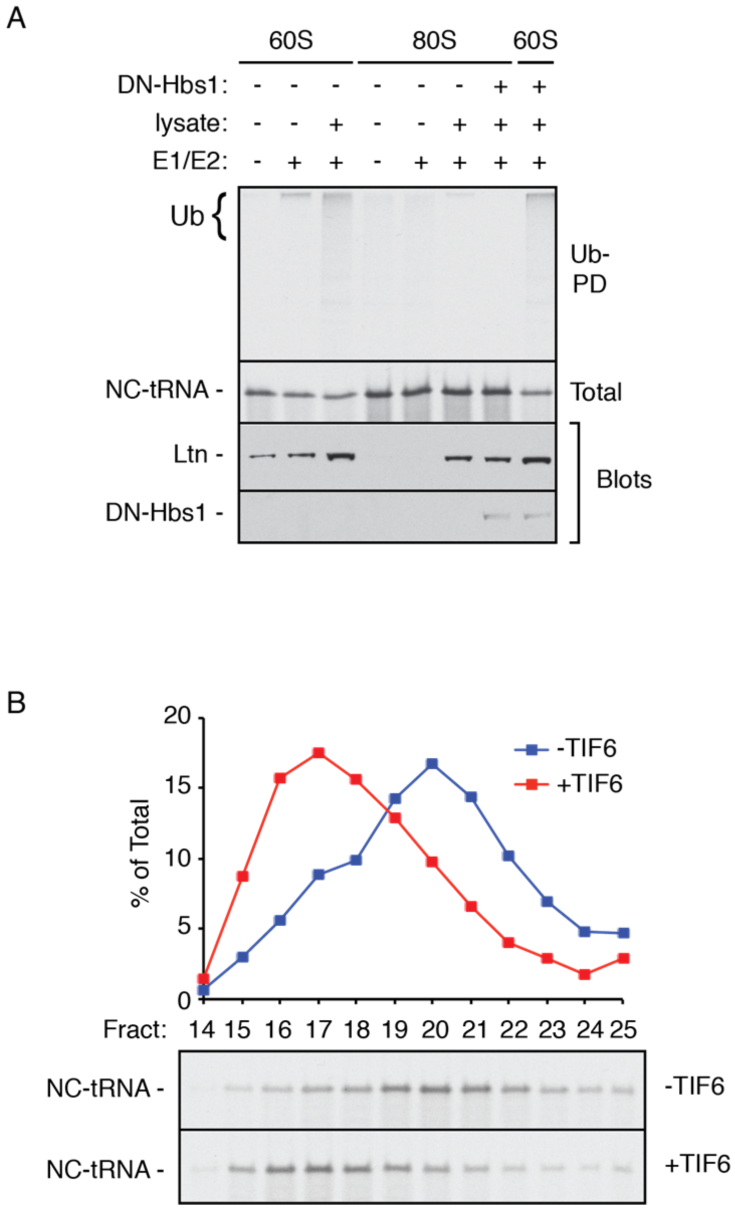
A
60S
80S
60S
DN-Hbs1: - - - - - - + +
lysate: - - + - - + + +
E1/E2: - + + - + + + +
Ub
Ub-PD
NC-tRNA -
Total
Ltn -
DN-Hbs1 -
Blots
B
% of Total
-TIF6
+TIF6
Fract: 14 15 16 17 18 19 20 21 22 23 24 25
NC-tRNA -
-TIF6
NC-tRNA -
+TIF6

Sup. Fig. S7 (related to Fig. 7). 80S RNCs are not efficient substrates for ubiquitination. (A) β-VHP RNCs were translated in lysate lacking the relevant E2 (see Fig. 4C) and separated on a 4.8 mL 10-30% sucrose gradient. 60S and 80S fractions were pooled and subject to ubiquitination reactions as in Fig. 4D in the presence or absence of S100 and/or DN-Hbs1. S100, despite containing Listerin, results in only a minor amount of ubiquitination of 80S-RNCs. This minor ubiquitination on 80S is completely inhibited by DN-Hbs1, suggesting that it occurred only after splitting into 60S-nascent chain complexes. Importantly, DN-Hbs1 does not affect the ubiquitination of 60S-nascent chains. **(B)** Duplicate translation reactions of β-VHP RNCs were initiated in RRL for 10 minutes, at which point the translation initiation inhibitor ATA was added. To one reaction, excess recombinant TIF6 was added to prevent re-association of any free 60S and 40S subunits. The reactions were then allowed to proceed to 45 minutes, separated on a 4.8 mL 10-30% sucrose gradient, and individual fractions analyzed by SDS-PAGE and autoradiography. Shown are fractions containing 60S and 80S particles. Note that in the presence of TIF6, the majority of the substrate is shifted to 60S fractions. TIF6 has been well-characterized to bind the 60S subunit to prevent rejoining with the 40S, but with no active effect on ribosome splitting. Thus, the observation that most stalled RNCs migrate as 80S complexes in the absence of TIF6 is probably explained by the rejoining of 40S subunits with 60S-NC-tRNA complexes after efficient splitting.

Supplemental Experimental Procedures

Plasmids and antibodies. The SP64 vector-based constructs encoding PrP, GFP, and Sec61β lacking the transmembrane domain and containing a C-terminal 3F4 epitope tag (Sec61β-ΔTMD) have been described (Kang et al., 2006; Stefanovic and Hegde, 2007; Sharma et al., 2010; Hessa et al., 2011). β-VHP-FL (Fig. 1A) was generated by inserting synthetic oligonucleotides encoding the 35 amino acid sequence of villin headpiece (LSDEDFKAVFGMTRSAFANLPLWKQQNLKKEKGLFGS; McKnight et al., 1996) into the Bsu36I restriction enzyme site between amino acids 14 and 15 of Sec61β-ΔTMD. For affinity purification, a 3X HA tag was inserted at the N-terminus using standard subcloning techniques. β-VHP-K12 was generated by inserting a 36-nt polyA oligonucleotide into the AgeI site of Sec61β-ΔTMD. A previously described stem-loop sequence (Doma and Parker, 2006) was inserted at the same site to generate β-VHP-SL. Pelota cDNA (Open Biosystems) was cloned into the pRSETA vector for bacterial expression and subsequent purification via the 6xHis-tag. Hbs1 cDNA (Open Biosystems) was cloned into a pcDNA3.1-based vector for mammalian expression and purification via an N-terminal 3X-FLAG tag. GTPase-deficient dominant negative Hbs1 constructs (V269G and H348A) were generated using standard mutagenesis techniques. The Anti-Ltn1 (Abcam), anti-Hbs1 (Abcam), anti-RPL9 (Santa Cruz), anti-RPS16 (Santa Cruz) antibodies were purchased. The anti-HA, anti-Sec61β, and anti-3F4 epitope antibodies were raised in-house against KLH-conjugated peptides. Anti-HA and anti-FLAG affinity resins were from Sigma.

Transcript preparation. All transcripts (except that coding for β-VHP-SL) utilized PCR products as templates for in vitro transcription (Sharma et al., 2010). The 5' primer anneals just before the SP6 start site in the SP64 vector. The 3' primer for making RNCs of β-VHP and ΔVHP-β anneals at codons 63-68 (using human Sec61 β numbering), which encodes nascent chains of 105 and 68 residues, respectively. For purification and crosslinking of dropoff nascent chain-tRNA complexes (Sup. Fig. S5), the 3' primer truncates the transcript after 40 residues to facilitate efficient drop-off. The 3' primer for β-VHP-FL and β-VHP-K12 anneals ~200 bp downstream of the stop codon. The 3' primer for PrP and GFP RNCs anneals to end at the last sense codon. β-VHP-SL transcripts were generated from plasmid linearized 180 nt after the stop codon. β-VHP-pA transcript was generated from β-VHP transcript by adding a PolyA tail using PolyA polymerase (NEB) at 37°C for 1 hr, followed by purification using the RNeasy Kit (Qiagen). Preliminary time course experiments established the conditions needed to generate ~200 nt polyA tails (verified by gel electrophoresis). ^{33}P-labeled transcripts were generated by incorporating ^{33}P-UTP (Perkin-Elmer) into transcription reactions and purifying the transcript with RNeasy Kit. Radiolabeled transcripts were included in translation reactions at a concentration that is 2% of the typical amount of transcript used.

***In vitro* translation.** *In vitro* translation reactions using rabbit reticulocyte lysate (RRL) were as described (Sharma et al., 2010). Translation systems utilizing phenyl-depleted RRL (Sup. Fig. S1) and DEAE fractionated RRL (Fr-RRL; Fig. 4C, 4D) were prepared as before (Hessa et al., 2011; Mariappan et al., 2010). The phenyl-depleted lysate lacks most major hydrophobic binding proteins, including the Bag6 complex, and is therefore incompetent for ubiquitination of their clients (Hessa et al., 2011). The Fr-RRL lacks ubiquitin and the Ubc4/5 family E2 enzymes. ΔHbs1 RRL (Fig. 6A, Sup. Fig. S6A) was

generated by incubating 800 μl of nuclease-treated RRL with 200 μl of packed Sepharose resin conjugated to recombinant Pelota purified from *E. coli*. Conjugation was via activated CnBr Sepharose (Sigma). Mock conjugated and quenched CnBr resin was used for control depletions. Depleted RRL was then reconstituted into a translation system as previously described (Sharma et al., 2010). Unless otherwise indicated, typical translation reactions were for 60 min at 32°C. To evaluate the effect of synthesizing less protein (Sup. Fig. S2E), the amount of transcript included in the translation reaction was diluted 2- to 100-fold by mixing with a mock transcription reaction containing no DNA. For experiments utilizing recombinant Hbs1, translation reactions were treated with 100 μM aurin tricarboxylic acid (ATA) at 10 min to prevent further initiation. Recombinant WT or dominant-negative (DN) Hbs1 were typically added at this time, after which reactions were allowed to proceed to 60 min. For direct analyses of ubiquitination and for the time course (Sup. Fig. S3A), translation samples were rapidly denatured in 1% SDS and heated to 100°C to stop all reactions. For applications requiring native complexes for downstream analysis, translation reactions were rapidly cooled on ice and used subsequently at 0-4°C for RNC isolation, sucrose gradients, and native immunoprecipitations (IPs) as described below.

Sucrose gradients and RNC treatments. For most sucrose gradient analyses, 200 μl translation reactions were cooled to 0°C and immediately layered onto 2-ml 10-50% sucrose gradients in RNC buffer (RB: 50 mM Hepes, pH 7.4, 100 mM KAc, 5 mM $MgCl_2$). 0.1% Triton X-100 was included in some experiments but this was without effect on the results. Centrifugation was in a TLS-55 rotor (Beckman) at 55,000 rpm for 1 hr at 4°C, using the slowest acceleration and deceleration settings, after which eleven 200 μl fractions were collected from the top. Each fraction was analyzed directly (indicated as 'total' in the figures), subjected to pulldowns using immobilized Ni^{2+} or Co^{2+} to isolate His-tagged ubiquitinated species, or subjected to IPs. The pulldowns and IPs were performed on samples that were first denatured in 1% SDS. In some experiments, fractions 5-7 containing the RNCs (as judged by A260 measurements) were pooled for downstream analyses. For RNase treatment (Fig. 1C, 2B and 2D), a final concentration of 50 μg/ml RNase A was added to pooled RNCs and incubated for 10 min at 37°C. To precipitate tRNA-associated species (Fig. 1C), a final concentration of 1% CTAB, 0.25 M sodium acetate was added to RNC fractions with 0.2 mg/ml tRNA as a carrier (Mariappan et al., 2010). Samples were incubated for 10 min at 32°C and then spun for 10 min at 13,000 rpm in a tabletop centrifuge. The pellets were dissolved in 1% SDS, 0.1 M Tris, pH 8.0 and either analyzed directly or subject to IPs with control or specific antibodies. The supernatants were precipitated with TCA for gel analysis. Puromycin treatment (Sup. Fig. S2A) was carried out with 1 mM puromycin at 32°C for 30 min. For higher-resolution gradients (e.g., Fig. 4), 200 μl translation reactions were layered onto 4.8-ml 10-30% sucrose gradients in RB and spun in a MLS-50 rotor (Beckman) at 50,000 rpm for 2 or 2.5 hr at 4°C with the slowest acceleration and deceleration rates. Twenty-five 200 μl fractions were collected from the top. These samples were used subsequently in ubiquitination assays (see below). For direct analysis of the gradient fractions, a portion of each fraction was combined with an equal volume of 50 mM Hepes, pH 7.4, 100 mM KAc, 5 mM $MgCl_2$, 1% Triton X-100 and subject to TCA precipitation.

RNC and NC-tRNA affinity purification. To affinity purify native RNCs (Sup. Fig. S2B), 3X HA-tagged VHP-β RNCs were translated and separated on a 10-50% sucrose

gradient in the presence of 0.1% Triton X-100. RNC fractions 5-7 were pooled, split into 250 µl aliquots and incubated with 20 µl of either control or anti-HA resin at 4°C for 1.5 hr. The resin was washed with 2ml RB with 0.1% Triton X-100, 2 ml of high salt buffer (50 mM Hepes, pH 7.4, 300 mM KAc, 10 mM MgCl2, 0.5% Triton X-100), 2 ml of RB with 0.1% Triton X-100, and eluted with protein sample buffer for direct analysis by SDS-PAGE. Native RNC isolation in Fig. 3F, 6D and Sup. Fig. S5B was performed directly from a translation reaction by diluting the sample 20-fold in 100 KAc, 25 mM Hepes, pH 7.4, 0.1% Triton X-100, and either 2 mM $MgCl_2$ or 10 mM EDTA as indicated. The samples were incubated with 20 µl of either control or anti-HA resin at 4°C for 1.5 hr. For Fig. 3F, the resin was washed three times in the same buffer as the incubation, and eluted with SDS-PAGE sample buffer. For Fig. 6D and Sup. Fig. S5B, the resin was washed with 2 ml of high salt buffer (25 mM Hepes, pH 7.4, 0.5% Triton X-100, 250 mM KAc, 2 mM $MgCl_2$), then 2 ml with the binding buffer, and eluted with SDS-PAGE sample buffer. For purification of drop-off nascent chain-tRNA complexes for mass spectrometry (Sup. Fig. S5A), a translation reaction of 3XFlag-tagged Sec61β-40mer was spun to remove ribosomes. The S100 supernatant was then split into 3 aliquots of 2 ml, of which one was treated with RNase. Each 2 ml aliquot was incubated with 50 µl of packed anti-Flag resin at 4°C for 1 hr in disposable columns. The resin was washed with 9 ml of 50 mM Hepes, pH 7.4, 100 mM KAc, 5 mM $MgCl_2$, 0.1% TX-100. One column of untreated S100 was first eluted with RNase (Sup. Fig. S5A, lane 2), and then with 3X Flag peptide (lane 1). The other two columns were directly eluted with 3X Flag peptide (lanes 3 and 4). Elutions were TCA precipitated before SDS-PAGE analysis.

Isolation of native and salt-stripped RNCs. To isolate native ('low salt') RNCs, 1 ml translation reactions were layered on a 1.6 ml 0.5M sucrose cushion in RB and centrifuged in a TLA-100.3 rotor (Beckman) at 100,000 rpm for 1 hr at 4°C. High-salt-stripped RNCs were generated the same way, except that 1 ml translation reactions were adjusted to a final concentration of 750 mM KAc, 15 mM $MgCl_2$ and layered on 1.6 ml 0.5M sucrose cushions in high salt buffer (50 mM Hepes, pH 7.4, 750 mM KAc, 15 mM $MgCl_2$). The cytosolic material and sucrose were aspirated, and the RNC pellets were resuspended in 50 µl of RB+10% sucrose. These were used immediately or flash frozen in liquid nitrogen for later use in ubiquitination reactions.

S-100 generation and depletion. S-100 cytosol was generated by centrifuging crude RRL (Green Hectares) in a TLA-100.3 rotor at 100,000 rpm for 1 hr at 4°C. The supernatant was recovered for downstream assays. To assay the effect of factor depletion by translating different substrates (Sup. Fig. S3D and S3E), non-radioactive mock or VHP-β translation reactions were centrifuged in a TLA-120.1 rotor at 85,000 rpm for 30 min at 4°C. The supernatant was recovered and used to assay for its ability to support ubiquitination. To immunodeplete listerin (Fig. 3C), 50 µg of anti-Ltn1 or control antibody was bound to 30 µl Protein A-agarose at 4°C for 2.5 hr. The resin was washed and incubated with 60 µl S-100 for 10 min at 4°C. The S-100 was passed over the resin several times and then incubated with fresh anti-Ltn1 or control Protein A resin at 4°C for 1 hr. The unbound material was collected for use in ubiquitination assays.

Ubiquitination assays. For analysis of ubiquitination that occurs in the translation reaction (e.g., Fig. 1C, 1D), 10 µM His-tagged or FLAG-tagged ubiquitin (Boston Biochem) was included to facilitate isolation of ubiquitinated products. To analyze the

effects of mutant ubiquitin (Sup. Fig. S2D), wildtype, K48R, and K0 His-tagged ubiquitin (Boston Biochem) was included during the translation reaction at a final concentration of 25 μM. To test the E2 requirement in Fr-RRL (Fig. 4C), the E2 UbcH5a (Boston Biochem) was included at a concentration of 250 nM during translation. Translation times in all of the above analyses were for 1 hr at 32°C. For post-translational ubiquitination assays on isolated RNCs (e.g., Fig. 3A), 20 μl reactions contained 2 μl of RNCs, 75 nM E1, 250 nM UbcH5a (both from Boston Biochem), an energy regenerating system (ERS: 1 mM ATP, 1 mM GTP, 12 mM creatine phosphate, 20 μg/ml creatine kinase), and 10 μM His-ubiquitin or FLAG-ubiquitin. For evaluation of cytosolic components (e.g., Fig. 3D), between 2 to 8 μl S-100 (immunodepleted in some cases) was included. Reactions were incubated at 32°C for 45 min, stopped by the addition of 1% SDS, and boiled. Samples were either analyzed directly or subject to pulldowns using either immobilized Ni^{2+} or Co^{2+} to isolate ubiquitinated species. For screening of E2 enzymes (Sup. Fig. S3C), a final concentration of 100 nM of each E2 was used in the reactions. Ubiquitination reactions using sucrose gradient fractions (e.g. Fig. 4C) were scaled up to 150 μl to accommodate dilution through the gradient. Final concentrations of E1, E2, ERS components, and His-ubiquitin remained the same as above.

Purification of recombinant proteins. Recombinant His6-tagged Pelota used to deplete Hbs1 (Fig. 6A, Sup. Fig. S6A) was expressed in BL21(DE3) pLysS *E. coli* and purified via the His tag on immobilized Ni^{2+} or Co^{2+}. The purified protein was conjugated to CnBr-activated sepharose (Sigma) according to standard procedures directed by the manufacturer. Flag-tagged recombinant wild type and dominant negative Hbs1 proteins (Fig. 6B-E, Sup. Fig. S6B-E) were transfected into HEK 293T cells. Transfected cells were maintained for 3 days and harvested in 50 mM Hepes, 150 KAc, 4 mM $MgAc_2$, 1% Triton X-100, 1 mM DTT, and protease inhibitor cocktail. The post-nuclear supernatant of the lysate was incubated with anti-Flag resin at 4°C for 1.5 hr. The resin was washed in the lysis buffer containing 400 mM KAc, and the protein was eluted with Flag peptide in 50 mM Hepes, 120 mM KAc, 2 mM $MgAc_2$, 1 mM DTT. Recombinant His6-tagged TIF6 (plasmid kindly provided by A. Warren, MRC) was expressed in BL21(DE3) pLysS *E. coli*, purified via the His tag on immobilized Co^{2+}, and dialyzed into 50 mM Hepes, pH 7.4, 150 mM KAc, 2 mM Mg Ac_2, 15% glycerol, 1 mM DTT for functional assays. A final concentration of 25 μg/mL of purified TIF6 was added 10 minutes after the start of a translation reaction to prevent ribosome subunit reassociation (Sup. Fig. S7B).

Tissue Culture Analyses. Actively growing HEK 293T cells (~50% confluent) in 6-well plates or 10 cm dishes were treated with either nothing (or a DMSO control), 200 nM pactamycin (PAC), or 50 μg/mL cycloheximide (CHX) for 1 hour (Fig. 3G and 4B). Cells were washed in PBS containing the same amount of treatment and the cytosol extracted with digitonin-containing buffer (25 mM Hepes, pH 7.4, 125 mM KAc, 15 mM $MgAc_2$, 100 μg/mL digitonin, 40U/mL RNase inhibitor, 50 μg/mL CHX, 1 mM DTT, 1X protease inhibitor cocktail) on ice for 5 min. The lysate was spun at maximum speed in a benchtop microcentrifuge for 10 min at 4°C , and the supernatant was separated either on a 2 mL 10-50% or a 4.8 mL 10-30% sucrose gradient as detailed above. Fractions were collected and measured for their absorbance at 260 nm or subject to TCA precipitation for analysis by SDS-PAGE and immunoblotting.

Miscellaneous biochemistry. Denaturing pulldowns of His-tagged ubiquitinated products were conducted as described previously with minor modifications (Hessa et al., 2011). Samples in 1% SDS were heated, diluted 1:10 with pulldown buffer (PBS containing 250 mM NaCl, 0.5% Triton X-100, 20 mM imidazole) and incubated with 10 µl of packed resin containing Ni^{2+} or Co^{2+} immobilized on chelating sepharose. Pulldowns of Flag-tagged ubiquitinated products were denatured in the same way and incubated with 10 µl of anti-Flag resin. Denaturing immunoprecipitations (Fig. 2D, Sup. Fig. S2F, S5C) were performed in the same way except that denatured samples were incubated with 1:330 of anti-Sec61β N-terminal or 3F4 antibody or 1:50 of anti-Listerin antibody in the presence of 10 µl packed Protein A resin. After 1-2 hr at 4°C, samples were washed two times with pulldown buffer and eluted directly in protein sample buffer (for Flag-pulldowns and IPs) or sample buffer containing 30 mM EDTA (for His-ubiquitin pulldowns) for SDS-PAGE analysis. SDS-PAGE was carried out on 8% or 12% tricine gels. For immunoblotting, samples were transferred onto 0.2 µm nitrocellulose membranes and blocked with 5% milk in PBS with 0.1% Tween. Primary antibodies were incubated at concentrations of 1:500 anti-Ltn1, 1:200 anti-Hbs1, and 1:100 anti-RPL9 and anti-RPS16. TCA precipitations were conducted on samples that typically contained 0.5% Triton X-100 as a carrier, to which 15-20% TCA was added. Samples were incubated on ice for 10 min, spun in a tabletop centrifuge at 13,000 rpm for 1 min, washed with 1 ml of cold acetone, allowed to air dry and resuspended in 0.1M Tris, 1% SDS or directly into protein sample buffer for SDS-PAGE analysis. Crosslinking of drop-off nascent chain-tRNA complexes (Sup. Fig. S5C) was done by separating a translation reaction of Sec61β-68mer on a 5-25% sucrose gradient at 55K rpm, 5 hr (TLS-55). Fractions 8-11 of a total of eleven 200 µl fractions were pooled and separated into three aliquots, one of which was treated with RNase. All three aliquots were then crosslinked with 250 µM of BMH for 30 min at room temperature (XL) before the reaction was quenched with 25 mM DTT. One of the crosslinked-only fractions was quenched in the presence of RNase. All samples were then denatured in 1% SDS for downstream analysis as described.
